# Nanodisc-cell fusion: control of fusion pore nucleation and lifetimes by SNARE protein transmembrane domains

**DOI:** 10.1038/srep27287

**Published:** 2016-06-06

**Authors:** Zhenyong Wu, Sarah M. Auclair, Oscar Bello, Wensi Vennekate, Natasha R. Dudzinski, Shyam S. Krishnakumar, Erdem Karatekin

**Affiliations:** 1 Department of Cellular and Molecular Physiology, School of Medicine, Yale University, 333 Cedar Street, New Haven, CT, 06520, USA; 2 Nanobiology Institute, Yale University, 850 West Campus Drive, West Haven, CT, 06516, USA; 3 Department of Cell Biology, School of Medicine, Yale University, 333 Cedar Street, New Haven, CT, 06520, USA; 4 Department of Molecular Biophysics and Biochemistry, Yale University, 260 Whitney Avenue, New Haven, CT 06520, USA; 5 Laboratoire de Neurophotonique, Université Paris Descartes, Faculté des Sciences Fondamentales et Biomédicales, Centre National de la Recherche Scientifique (CNRS) UMR8250, 45, rue des Saints Pères, 75270 Paris Cedex 06, France

## Abstract

The initial, nanometer-sized connection between the plasma membrane and a hormone- or neurotransmitter-filled vesicle –the fusion pore– can flicker open and closed repeatedly before dilating or resealing irreversibly. Pore dynamics determine release and vesicle recycling kinetics, but pore properties are poorly known because biochemically defined single-pore assays are lacking. We isolated single flickering pores connecting v-SNARE-reconstituted nanodiscs to cells ectopically expressing cognate, “flipped” t-SNAREs. Conductance through single, voltage-clamped fusion pores directly reported sub-millisecond pore dynamics. Pore currents fluctuated, transiently returned to baseline multiple times, and disappeared ~6 s after initial opening, as if the fusion pore fluctuated in size, flickered, and resealed. We found that interactions between v- and t-SNARE transmembrane domains (TMDs) promote, but are not essential for pore nucleation. Surprisingly, TMD modifications designed to disrupt v- and t-SNARE TMD zippering prolonged pore lifetimes dramatically. We propose that the post-fusion geometry of the proteins contribute to pore stability.

All membrane fusion reactions necessarily involve an initial, narrow connection between the fusing membranes called the fusion pore^1^. Fusion pores have been observed during hormone^1^ and neurotransmitter release^2^,^3^,^4^, cell-cell^5^,^6^,^7^ and cell-artificial bilayer fusion^8^ induced by viral proteins expressed on cell surfaces, and for bilayer fusion in the absence of any protein^9^. In all cases, a fraction of the pores flickered between open and closed states multiple times before either dilating (leading to full fusion) or resealing irreversibly (resulting in transient fusion). For hormone secretion, pore dynamics are physiologically regulated and determine the amount and kinetics of release, and the mode of vesicle recycling^10^. In addition, fusion pores may act as size-selective filters through which only small cargo can escape^10^. Neurotransmitters can also be released through flickering fusion pores^2^,^3^,^4^, with important consequences for downstream events such as the speed of vesicle recycling or receptor activation^4^,^11^,^12^. Despite being a key intermediate for all fusion reactions, factors controlling nucleation and dynamics of fusion pores are poorly understood, in part due to a lack of suitable methods to probe them.

Electrophysiological, electrochemical, and optical methods have been applied to study fusion pores, mostly for calcium-triggered exocytosis which underlies neurotransmitter and hormone release^11^,^13^,^14^. Although electrical and electrochemical approaches provide the most direct readout of fusion pore dynamics, such methods have been difficult to apply to reductionist systems which are nevertheless required to deduce molecular mechanisms governing pore nucleation and dynamics. Optical methods, in contrast, have been abundantly applied to study fusion of liposomes with other liposomes in bulk^15^, single liposome–liposome^16^ or single liposome–supported bilayer fusion^17^,^18^,^19^, and most recently for bulk nanodisc–liposome fusion^20^,^21^. The most quantitative information about fusion pore dynamics that can be extracted, however, is currently limited to a time-averaged pore openness^22^. We therefore developed a novel assay to probe single fusion pore dynamics with sub-millisecond time resolution in a biochemically defined setting. We have applied the method to study fusion pores induced by the core components of the exocytotic/neuronal fusion machinery, the *soluble N-ethylmaleimide-sensitive factor attachment protein receptors* (SNARE) proteins.

Most intracellular fusion reactions, including calcium-triggered release of neurotransmitters and hormones, are driven by pairing of vesicle-associated v-SNAREs with cognate t-SNAREs on the target plasma membrane^23^. Complex formation between the neuronal/exocytotic v-SNARE *vesicle-associated membrane protein 2* (VAMP2, also known as synaptobrevin-2) and the t-SNAREs syntaxin-1 (Stx1) and *synaptosomal-associated protein 25* (SNAP25) starts from the membrane distal N-termini, proceeding in stages^24^ toward the membrane proximal regions, resulting in a four-helix bundle (SNAREpin) that brings bilayers into close proximity. However, it is not known how a pore nucleates at this stage. There are at least two mechanisms that could contribute to pore nucleation. First, continued SNARE assembly through the transmembrane domains (TMDs) may drive pore opening, as suggested by a recent crystal structure^25^ of the neuronal SNARE complex that showed multiple contacts between the v- and t-SNARE TMDs, and the observation that mutations of VAMP2 TMD reduced exocytosis in a secretory cell line^26^. Second, the TMDs may act as passive anchors pulled by SNAREpins as they assemble to force the membranes close together^27^,^28^, because replacing the TMDs with lipid anchors does not abolish fusion, provided the lipid anchor spans both leaflets^27^ or consists of multiple single-leaflet spanning acyl chains^28^,^29^. Since the hydrophobic TMDs are expected to pack tightly in micelles used for crystallization^25^, the crystal structure contacts may be due to packing constraints. Distinguishing between these possibilities has proven difficult using conventional methods. Using the new single fusion pore assay, we show that interactions between v- and t-SNARE TMDs promote, but are not essential for pore nucleation. Surprisingly, disrupting the putative v- and t-SNARE TMD interactions dramatically prolonged pore lifetimes.

## Results

### Detection of single fusion pores connecting nanodiscs to cells

**Figure 1:**
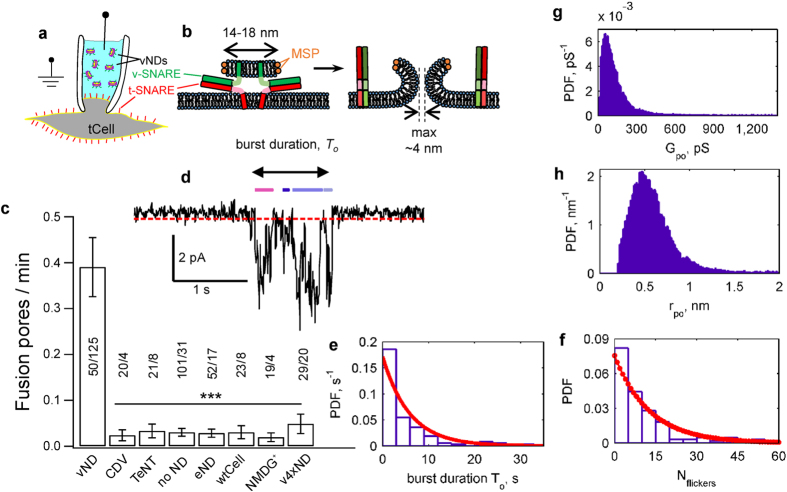
Detection of single fusion pores between v-SNARE nanodiscs (vNDs) and flipped t-SNARE cells (tCells). (**a,b**) Schematic of the setup and vND-tCell fusion. A pipette is sealed onto the tCell to isolate a patch of membrane (**a**). Unlike fusion of a closed vesicle with a voltage-clamped membrane, vND-tCell fusion establishes a direct ionic conduction pathway between the cell cytosol and the pipette solution (**b**). The schematic in b is approximately to scale. (**c**) Fusion pores are SNARE-induced. When the soluble cytoplasmic domain of the v-SNARE VAMP2 (CDV), or the tetanus neurotoxin light chain (TeNT) was included in the pipette solution, currents occurred much less frequently. Similarly, not including NDs in the pipette (no ND), using SNARE-free, empty NDs (eND), wild-type cells not expressing flipped t-SNAREs (wtCell), replacing Na^+^ with the larger NMDG^+^, or replacing wild-type VAMP2 with a mutant that was previously shown to induce docking but not fusion largely abolished currents. (***Indicates p < 0.001, t-test against vND-tCell). The number of patches/pores for each condition is indicated. (**d**) Example of a fusion pore current “burst”. Open sub-states are defined as having current <−0.25 pA (red dashed line) for at least 60 ms. Colored bars above the fusion pore indicate detected open periods. (**e**) Distribution of burst lifetimes, *T*_*o*_, as defined in (**d**). The red, solid line is an exponential fit, with mean 5.8 s. (**f** ) Distribution of flicker numbers 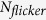, and fitted geometric distribution (red solid line, *y* = *p*(1 − *p*)^*n*^*, n* = 0, 1, 2, 3, …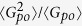 with *p* = 0.754 ± 0.0129 (95% confidence interval). (**g**) Probability density function of open-pore conductances, *G_p_*_*o*_, averaged over 122 individual fusion pores from 50 cells. (**h**) Probability density function for open-pore radii. For (**e–h**), the mean ± S.E.M. were *T*_*o*_ = 5.8 ± 0.9 s, *N*_*flicker*_ = 12 ± 2, *G*_*po*_ = 152 ± 12 pS, and *r*_*po*_ = 0.60 ± 0.02 nm.

To detect single fusion pores using electrophysiological recordings, we adapted tools that had been developed previously, namely lipid bilayer nanodiscs (NDs) bearing neuronal/exocytotic v-SNAREs^20^ and “tCells” expressing engineered cognate “flipped” t-SNAREs whose topology is inverted, with the active SNARE domains facing the extracellular space rather than the cytosol^30^,^31^,^32^. Unlike a vesicle, a ND is not a closed structure: its fusion with the cell membrane establishes an ion-conduction pathway between the cytosol and the extracellular space, allowing direct-current measurements to reveal single pore properties with sub-millisecond time resolution. We modified the classic cell-attached voltage-clamp configuration^33^ as shown in Fig. 1. We filled the tip of the pipette with ~1 μl buffer, and overlaid on top a solution containing 16 ± 2 nm diameter bilayer discs stabilized by the membrane scaffold protein (MSP)^20^, each bearing 7–9 copies of the v-SNARE VAMP2^20^. This arrangement provided enough time for tight seal formation and recording a stable baseline under voltage-clamp (16 mV across the patch) before the v-SNARE discs (vND) diffused to the patch membrane and started fusing with it 13 ± 6.6 min (mean ± S.E.M.) after seal formation (Supplementary Fig. 1). Fluorescently labelled discs reach the tip with a characteristic time of 7–9 min (Supplementary Fig. 1c), indicating a significant delay between arrival and fusion of vNDs, consistent with a slow docking rate^20^. The initial few current bursts were well-separated in time, with typically >50 s between them (Supplementary Fig. 1d). Together with small unitary conductances (Fig. 1g), this strongly suggests each burst was due to a single vND fusing with the patch. The burst frequency increased, evidently due to arrival of more vNDs to the patch as time passed (Supplementary Fig. 1c,e). Quantification of the surface density of t-SNAREs indicated each patch contains 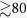 copies on average (Methods), which should allow at least several fusions before the t-SNAREs are consumed. Ignoring closely spaced or overlapping fusion pore currents, on average 0.39 ± 0.06 fusion pores per min (mean ± S.E.M.) were recorded. This underestimated fusion pore initial nucleation rate was compared with negative controls (Fig. 1c). Including the soluble cytoplasmic domain of VAMP2 (CDV) in the pipette solution as a competitor, treating the vNDs with the tetanus neurotoxin which cleaves VAMP2, using SNARE protein-free, or empty NDs (eND), or wild-type cells lacking flipped t-SNAREs (wtCell) resulted in >10 fold reduction in the rate of fusion pore-like currents, establishing that vND-tCell fusion is SNARE-dependent.

To test the possibility that the amphiphilic MSP in the NDs might have a role in pore nucleation, we patched tCells as above, but did not include any NDs in the pipette solution. Background currents obtained in this configuration occurred with the same low frequency as the other negative controls, with ~1 event for 2000 s of recording time (Fig. 1c, no ND). Is it still possible that the role of the SNAREs is limited to docking the NDs onto the plasma membrane, and the pores are nucleated by the MSP now in close proximity to the plasma membrane, rather than by the SNAREs? To rule out this possibility, we used a previously characterized v-SNARE construct bearing mutations in the C-terminal hydrophobic layers (L70D, A74R, A81D, and L84D, termed VAMP2-4X)^34^,^35^. The N-terminal half of this construct zippers and forms a stable complex with the t-SNAREs syntaxin1 and SNAP25, but the C-terminal half is prevented from zippering^34^,^35^. As a consequence, the construct allows efficient docking^34^, but is fusion incompetent^35^. If the role of the SNAREs were limited to bringing the MSP near the plasma membrane, we would expect a significant increase in the fusion rate using this construct compared to using empty NDs. We reconstituted VAMP2-4X into NDs (v4xNDs), and used these in the single-pore assay with tCells as fusion partners. Pore nucleation rate was not significantly different under these circumstances than any of the other negative controls (Fig. 1c), ruling out a role for MSP in pore nucleation.

Pore openings resulted in bursts of currents that fluctuated around 0.8–15 pA and lasted 5.8 ± 0.9 s (mean ± S.E.M.) on average (Fig. 1d,e and Supplementary Fig. 2. See Methods for detection criteria). Fluctuations around the mean open-pore currents exceeded baseline root mean-squared noise 5.5 ± 0.5 fold (mean ± S.E.M.), suggesting they were due to fluctuations in the pore geometry and/or unresolved flickers, similar to fluctuations often seen in exocytotic fusion pore conductances^3^,^36^ or amperometric currents^37^,^38^. Pores opened and closed (flickered) 12 ± 2 times (mean ± S.E.M.) during a burst before permanently resealing, and flicker numbers distributed geometrically (Fig. 1f), analogous to a single channel making discrete transitions between open, transiently blocked, and shut states^33^. Lifetimes of open and closed sub-states during a burst distributed exponentially (Supplementary Fig. 3). The pore open probability during a burst, calculated from the fraction of time a pore was in the open state during a burst, was *P*_*o*_ = 0.67 ± 0.02 (mean ± S.E.M.).

The distribution of open-pore conductances is broad, as shown in Fig. 1g, with mean 〈*G*_*po*_〉 = 152 pS, well within the values measured for fusion pores in secretory cells^13^. The pore radii (Fig. 1h), estimated from conductance^39^, spanned ~0.2 nm (size of a hydrated Na^+^ ion^39^) to ~2 nm, the maximum size we expected from the geometry of the largest MSP NDs^20^ (Fig. 1b). The mean open-pore radius, averaged over individual fusion pores, was 〈*r*_*p*o_〉 = 0.60 nm. In accord with this estimate, replacing Na^+^ in the pipette solution with N-methyl-D-glucamine (NMDG^+^), a large ion ~1.1 × 0.5 nm in size without its hydration shell^40^, reduced the fusion pore currents to baseline (Fig. 1c). Like exocytotic fusion pores that display broad distributions of conductances and lifetimes, individual fusion pores between vNDs and tCells also showed considerable variability (Supplementary Fig. 4).

**Figure 2:**
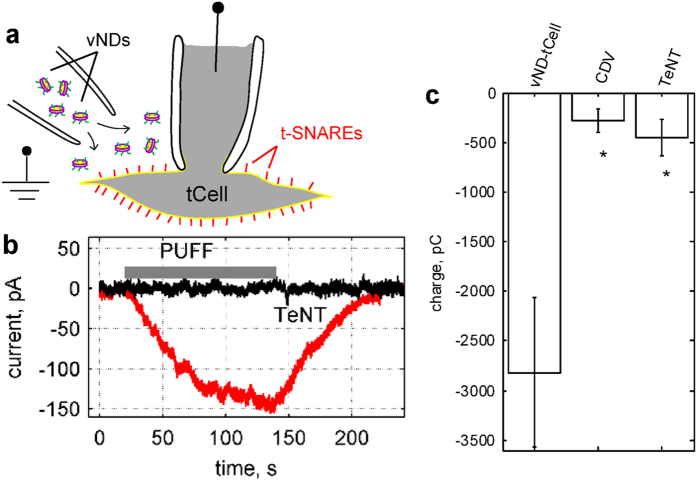
Fusion of vNDs with tCells elicits whole-cell currents. (**a**) Schematic of the setup. A tCell is voltage-clamped at −70 mV in the whole-cell configuration and vNDs are puffed nearby from a pipette. (**b**) Fusion of vNDs elicited whole-cell currents that were much larger than the unitary fusion pore currents obtained in the cell-attached configuration depicted in Fig. 1. Application of vNDs in the presence of TeNT did not elicit appreciable currents. (**c**) Whole-cell charge transfer that resulted from application of vNDs alone, or vNDs in the presence of CDV or TeNT. (n = 8, 7, and 4 cells for vND alone, and with CDV and TeNT). (*Indicates p < 0.05, t- test against vND alone.)

To ensure the currents we observed were due to fusion pores and not to cell-attached recording artefacts, we filled pipettes with an intracellular solution, whole-cell voltage-clamped tCells at −70 mV, and puffed vNDs through another pipette in close proximity (Fig. 2). This resulted in whole-cell currents of 156 ± 28 pA (mean ± S.E.M.), corresponding to a conductance ~15-fold larger than the average single-pore conductance, with some step-wise increments that could be discerned. Upon cessation of vND application, currents returned to baseline within 1–2 min.

Although we had a limited number of single-pore events for negative controls, we compared their characteristics with events obtained using NDs loaded with wild-type SNAREs (vNDs). When NDs were omitted from pipettes, pores lasted much shorter and flickered less compared to all other cases (Supplementary Fig. 5), suggesting the molecular architecture of these pores were different. It is possible that these pores that appeared very infrequently are simple bilayer pores^40^,^41^ or defects. Pore properties were indistinguishable in the presence of empty NDs or NDs loaded with wild-type or VAMP2-4X v-SNAREs. This suggests that randomly and infrequently appearing bilayer pores/defects may interact with NDs in a SNARE-independent manner and convert into fusion pores connecting ND and cell membranes whose properties are largely determined by those of lipid bilayers. It is well-known that free bilayer edges are highly “reactive”, tending to close pores^41^,^42^ and connecting to bilayer structures in close proximity^43^, analogous to the reactive ends of worm-like micelles that recombine rapidly^44^. Given the high density of NDs in the patch pipette (~0.15 μM after complete mixing), it would take 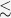
100 μs for a ND to collide with such a bilayer defect. The contribution of this SNARE-independent pathway for pore nucleation appears to be <10% according to the data in Fig. 1c. Nevertheless, we wanted to explore in more depth the possible contribution of MSP or other factors to pore nucleation and dynamics, as discussed below.

### Stretch-activated channels do not contribute to currents observed during vND-tCell fusion

HeLa cells have endogenous stretch-activated, non-selective, Mg^2+^ and Gd^3+^-sensitive cation channels^45^. To test if activity of such channels contributed to currents observed during vND-tCell fusion, we patched tCells and recorded cell-attached currents in the absence of NDs, varying the tension in the membrane patch by applying controlled suction through a high-speed pressure-clamp device (Supplementary Fig. 6). In the absence of Mg^2+^ and Gd^3+^, we observed stretch-activated currents only when suction pressure was >20 mmHg. The open probability of these channels increased as we increased suction (Supplementary Fig. 6a). The patches became fragile and broke more easily as suction was increased beyond 70 mmHg. Inclusion of 1 mM Mg^2+^, and especially of 30 μM Gd^3+^ largely suppressed these currents, consistent with a previous report^45^. From the fraction of time channels were open during a recording, we calculated the pore open probability at various suction pressures (Supplementary Fig. 6b). Because our vND-tCell fusion measurements were carried out without any suction (0 mmHg) in the presence of 1 mM Mg^2+^, contribution of stretch-activated channels should be negligible.

Could local stresses introduced by SNARE-mediated docking of vNDs in the vicinity of a stretch-activated channel cause channel opening? Our experiments using the docking-competent, fusion-incompetent VAMP2-4X argues against this possibility, because the frequency of currents did not significantly increase from background levels measured in the absence of NDs (Fig. 1c). In addition, currents due to stretch-activated channels (Supplementary Fig. 6) look very different from currents passing through fusion pores (Fig. 1d and Supplementary Fig. 2).

### Single-cell lipid mixing assay

**Figure 3:**
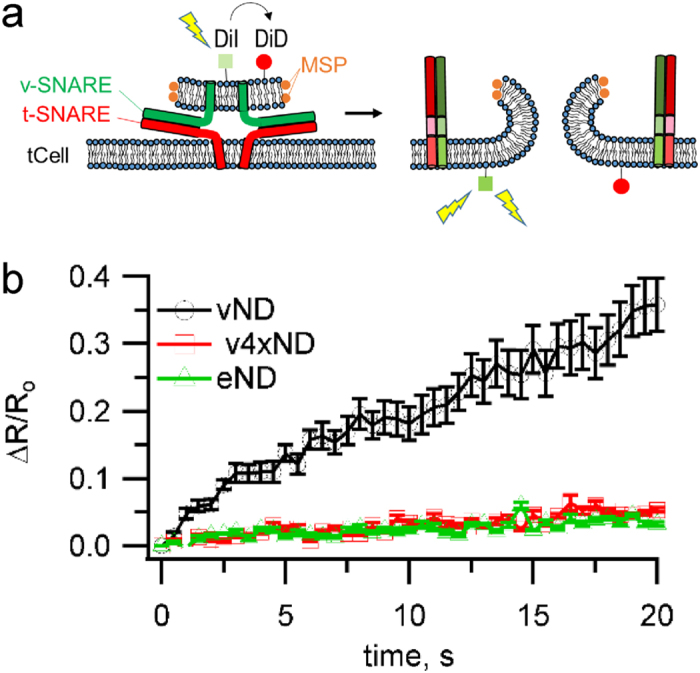
Single-cell lipid mixing assay. (**a**) Schematic of the approach. NDs containing 1% each of DiI and DiD lipid labels were pre-incubated with cells at 4 °C for 30 min to allow docking, but no fusion, with tCells. After rinsing twice with ice cold PBS to remove free NDs, PBS pre-heated to 37 °C was added and image acquisition was started and continued for 20 min using a spinning disc confocal microscope equipped with a temperature controlled stage set to 37 °C. For each image cycle, one frame recorded DiI fluorescence excited at 561 nm and the subsequent frame recorded DiD fluorescence excited at 647 nm. DiI fluorescence reports lipid mixing; upon fusion DiI and DiD are diluted in the plasma membrane and DiI is no longer quenched by DiD. At the labelling density used, DiD is not significantly self-quenched, so the DiD signal is proportional to the initial density of docked NDs. The ratio R of DiI-to-DiD fluorescence normalizes fusion signals for variations in docked ND density, temperature-induced fluorescence changes, and other instrumental and environmental factors. (**b**) Changes in DiI-to-DiD fluorescence ratio (ΔR), relative to the initial ratio (R_o_), for SNARE-free NDs (eND), or NDs loaded with wild-type (vND) or docking-competent, fusion-incompetent VAMP2-4X (v4xND). 11, 8, and 6 dishes were analysed for vND, v4xND and eND conditions, respectively.

To probe mixing of ND and cell membrane lipids, we incorporated the fluorescently-labelled lipids DiI and DiD at 1 mole % each into the same ND population. In separate experiments, we determined that at these concentrations, DiD fluorescence is only slightly (~10%) self-quenched, while DiI fluorescence is quenched >20-fold by fluorescence energy transfer to DiD. Thus, direct excitation of DiD fluorescence can be used as a docking signal, whereas an increase in DiI fluorescence as the dyes diluted in the cell membrane would report lipid mixing. To normalize the amount of lipid mixing to the amount of docked NDs per cell, we used the ratio of the DiI to DiD fluorescence intensities. We first incubated DiI-DiD co-labeled NDs (~90 nM ND, 36 μM lipid) with tCells adhering onto a glass-bottom dish at 4 °C for 30 min, a protocol that allows docking, but not fusion^15^. We then rinsed away free NDs before starting to monitor fluorescence signals at 37 °C using a confocal microscope. We alternated excitation of DiI (561 nm) and DiD (647 nm) fluorescence in successive frames, and monitored how these signals changed over time (Fig. 3). Empty discs, or discs loaded with VAMP2-4X did not produce any significant changes in the DiI/DiD fluorescence ratio over time. In contrast, the fluorescence ratio increased ~35% for NDs bearing wild-type v-SNAREs within 20 min, demonstrating efficient lipid mixing. We also used a slightly different protocol which avoided the cold pre-incubation step, but did not allow time-lapse imaging. After incubating the NDs with tCells for 15 min at 37 °C, we rinsed unbound discs and imaged tCells for a single DiI-DiD fluorescence cycle. Comparing the DiD fluorescence for the three conditions indicated that VAMP2-4X was just as efficient as wild-type VAMP2 in docking NDs onto tCells, whereas empty NDs were much less efficient (Supplementary Fig. 7b). The DiI-to-DiD fluorescence ratio corroborated the results obtained in time-lapse measurements (Supplementary Fig. 7c).

### Single-cell calcium influx assay

To ensure the currents we measured in patch-clamp experiments were due to currents passing through pores connecting NDs and cells rather than an artefact related to the method (such as the additional tension that exists in membrane patches isolated by a pipette), we developed an independent, fluorescence-based contents mixing assay. We loaded cells with Fluo-4, a calcium indicator whose fluorescence increases >100 fold when it binds Ca^2+^ (K_d_ = 0.3–1 mM)^46^. After washing away Fluo-4 remaining in the medium, we introduced NDs into the extracellular space (~90 nM NDs, 36 μM lipid) and monitored changes in Fluo-4 fluorescence using a spinning disc confocal microscope. When we used tCells and NDs bearing wild-type v-SNAREs, Fluo-4 signals increased (Supplementary Fig. 8), indicating extracellular calcium now had access to the cells’ interior. In contrast, when we used VAMP2-4X-loaded or empty discs, Fluo-4 signals did not change significantly, corroborating our findings in the single-pore experiments.

### Fusion pores connecting vNDs and tCells allow passage of neurotransmitter-like molecules

Our results above indicate fusion pores connecting NDs to cells allow flux of Na^+^ and Ca^2+^ through them, but restrict passage of NMDG^+^, a sugar derivative that is somewhat larger than many common neurotransmitters. We wondered if a probe molecule more similar to a neurotransmitter would be able to pass through the artificial fusion pores created in our experiments. For this, we incubated tCells in the presence of FFN202, a fluorescent false neurotransmitter^47^, for 20 min at 37 °C, in the absence of NDs, or the presence of eNDs or vNDs. We imaged the FFN202 fluorescence that remained after rinsing the cells, and subtracted the background signals obtained prior to FFN202 incubation. As shown in Supplementary Fig. 9, more FFN202 fluorescence remained in cells when vNDs were used compared to when NDs were omitted or eNDs used, consistent with FFN202 being taken up into the cells through fusion pores.

### Docking and fusion of vNDs with tCells are SNARE mediated

We used a toxin cleavage assay to probe whether docking was SNARE-mediated and if any post-fusion *cis*-SNARE complexes were found on the cell surface. These experiments indicated docking is mediated by v- and t-SNARE interactions in *trans*, followed (in the case of WT VAMP2) by fusion that produces toxin-resistant *cis*-SNARE complexes in the plasma membrane (Supplementary Note and Supplementary Fig. 10).

### Interactions between v- and t-SNARE TMDs promote, but are not essential for, pore nucleation

**Figure 4:**
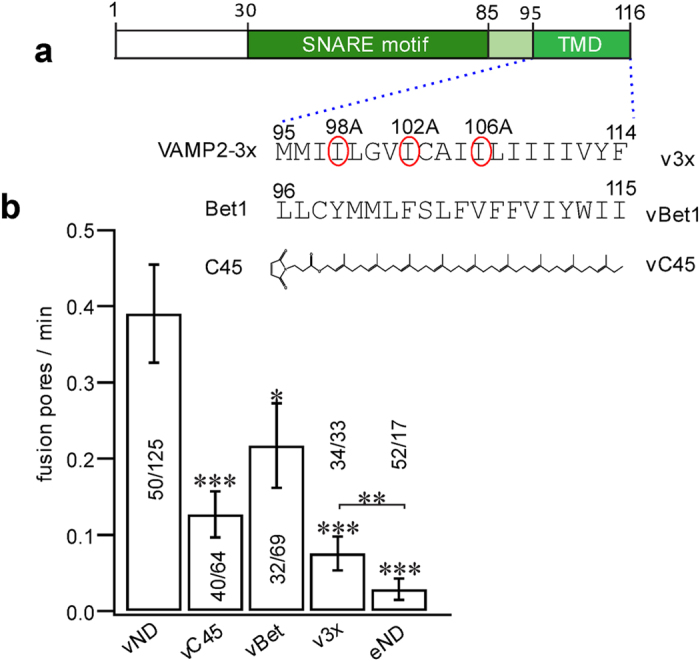
Zippering of v- and t-SNARE TMDs boosts pore nucleation but is not essential for it. (**a**) Schematic of the VAMP2 modifications used. VAMP2 TMD residues I98, I102, and I106 that contact syntaxin1 TMD residues in a crystal were mutated to alanines that readily incorporate into α-helices (v3x). Alternatively, the TMD of VAMP2 was replaced with that of Bet1 (vBet1), or a lipid anchor long enough to span the entire bilayer (27) (maleimidopropionic acid solanesyl ester) (vC45). (**b**) The rate of pore nucleation was significantly reduced for all TMD-modified v-SNAREs, but still larger than the rate with SNARE-free NDs (eND). The number of patches/pores for each condition is indicated. (*, **, and ***indicate p < 0.05, p < 0.01, and p < 0.001, respectively, using the t-test against vND.) vND and eND data are copied from Fig. 1c for comparison with data from TMD-modified SNAREs.

We used the new single-pore assay to test the functional role of putative v- and t-SNARE TMD interactions. For this, we selectively disrupted three of the 4–5 contacts indicated by a crystal structure^25^ by mutating isoleucines to alanines (v3x), or replaced the TMD of VAMP2 with a lipid anchor (vC45) or with that of Bet1 (vBet1), a distantly related v-SNARE involved in the endoplasmic reticulum to Golgi transport (Fig. 4a). A lipid anchor spanning both leaflets was chosen to maintain the anchor length about the same as the length of the TMD anchors, and because shorter anchors were not expected to result in compete fusion based on previous work^27^,^29^,^48^. All manipulations disrupting TMD-zippering resulted in markedly reduced nucleation rates compared to wild-type, but still significantly higher than for SNARE-free NDs (Fig. 4b). Because the cytosolic domains mediating docking are identical in all cases, these results suggest that v- and t-SNARE TMD interactions facilitate, but are not essential for pore nucleation after docking.

### Pore lifetimes are very sensitive to interactions between v- and t-SNARE TMDs

**Figure 5:**
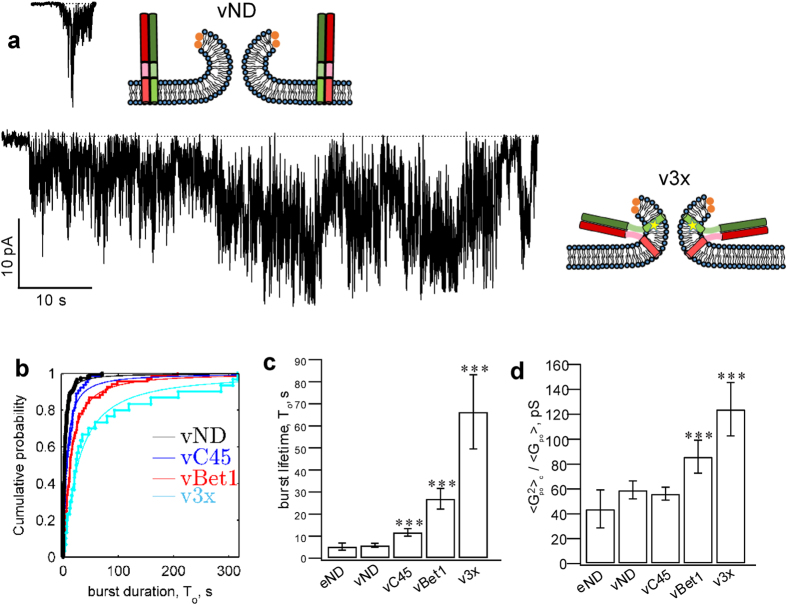
Geometric constraints may contribute to pore lifetimes. (**a**) Representative pore currents for NDs loaded with wild-type (vND) or v3x mutant VAMP2 (v3xND). Hypothetical post-fusion geometries of the VAMP2 proteins complexed with t-SNAREs are shown next to each trace. (**b,c**) Distribution of burst lifetimes (**b**) and their averages (**c**). Exponential fits (continuous lines) to data (staircase plots) are shown in (**b**). (**d**) Open-pore conductance fluctuations (variance) relative to mean, 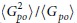. TMD-modifications that reduced TMD zippering stabilized pore lifetimes. (***Indicates p < 0.001, using the 2-sample Kolmogorov-Smirnov test against eND). The number of patches/pores for every condition as in Fig. 4.

Surprisingly, all manipulations aimed to disrupt the putative v-/t-SNARE TMD interactions resulted in 2 to 10-fold longer pore lifetimes compared to those obtained with wild-type SNAREs (Fig. 5).

When we used a previously described bulk assay probing calcium release during fusion between vNDs and t-SNARE reconstituted liposomes^20^, fusion rates scaled with the single-pore lifetimes of the TMD-modified v-SNAREs (Supplementary Note and Supplementary Fig. 11). In addition to fusion pore lifetime, conductance fluctuations relative to mean were significantly larger for vBet1ND and v3xND compared to wild-type and lipid anchored v-SNAREs (Fig. 5d). This may reflect changes in pore geometry as the TMDs of these mutants made repeated, unsuccessful attempts to zipper. Mean open-pore conductance, pore size, and pore open probability during a burst were not significantly different among the tested vNDs (Supplementary Fig. 12), indicating the major effect of SNARE complexes lingering at the fusion site is to obstruct irreversible pore closure, allowing more charge to be transferred per fusion pore (Supplementary Fig. 11).

### Initial pore expansion occurs in <1 ms

To probe the kinetics of the initial pore expansion, we aligned single fusion pore conductances to the first detected sub-opening in every burst and averaged them (Supplementary Fig. 13a). We found that pore opening proceeded with a rapid upstroke with a rise time of 3–9 ms, limited by how well the alignment could be made and our effective time resolution after signal processing (125 Hz bandwidth), for all cases tested. A 10-fold improvement in time-resolution (1.25 kHz bandwidth) also failed to resolve any differences in initial pore opening kinetics between pores induced by wild-type or v3x v-SNAREs (Supplementary Fig. 13b). This contrasts with the lower pore nucleation rates observed upon disrupting TMD-zippering (Fig. 5), and could either reflect our limited time resolution, or that the SNAREs and their TMDs mainly affect the probability of nucleating a pore, but not the initial expansion to *G*_*po*_ ≈ 100 pS (*r*_*p*o_ ≈ 0.5 nm), which may be limited by lipid rearrangements.

Interestingly, for both the lower and higher time-resolution data, a slowly rising baseline prior to the upstroke indicated many sub-threshold pore opening attempts preceded the first above-threshold opening by 100–400 ms (Supplementary Fig. 13). During calcium-triggered exocytosis, presumably the same machinery that prevents premature fusion from occurring at low calcium^49^ would also prevent such small pores that are barely permeable to small ions and provide a more rapid pore dilation upon calcium-induced activation.

## Discussion

The exquisitely detailed picture available about how the soluble domains of cognate v- and t-SNAREs assemble^24^ contrasts sharply with our blurry view of how the fusion pore opens and evolves during exocytosis, and its dynamic molecular composition. Existing methods have provided limited insight into these fundamental questions. On the one hand, properties of single exocytotic fusion pores have been characterized in settings where the biochemistry could not be controlled^1^,^13^. On the other hand, when biochemistry was well-controlled in reductionist systems, single fusion pores could not be probed for a lack of suitable approaches. As a consequence, there is a wealth of information accumulated from both types of approaches, but it is difficult to make connections between the two. Aiming to close this gap, we developed a single-pore assay that uses biochemically defined components. We used v-SNARE reconstituted nanodiscs (vNDs)^20^ and flipped t-SNARE cells^30^,^31^,^32^ ectopically expressing t-SNAREs on their outer surfaces as fusion partners. We voltage-clamped a tCell plasma membrane patch in the cell-attached configuration, and included vNDs in the pipette solution to record currents passing through single fusion pores as vNDs fused with the tCell surface.

Our results indicated SNAREs competent for complete zippering produce pores that are permeable to ions and neurotransmitter-like molecules. Production of these pores is accompanied by lipid mixing. A SNARE mutant defective in C-terminal zippering, VAMP2-4X, efficiently docks NDs onto tCells, but is able to produce neither lipid nor contents mixing. The amphiphilic MSP of the NDs, even when brought into very close proximity of the cell membrane by the fusion-incompetent VAMP2-4X, is evidently also unable to induce contents or lipid mixing. After fusion, VAMP2 is found in the cell membrane in a complex with the t-SNAREs in the post-fusion *cis* configuration. Interference of endogenous stretch-activated channels with fusion pore currents is negligible, and the additional membrane tension inherent in cell-attached patches is not required for fusion pore opening. Taken together, these results firmly establish NDs as fusion partners with flipped t-SNARE cells. This builds upon previous work that had established NDs as fusion partners with t-SNARE reconstituted small liposomes in a bulk assay^20^,^21^.

Our single fusion pore measurements directly revealed a facilitative, but non-essential role for v- and t-SNARE TMD interactions in pore nucleation. Thus, v- and t-SNARE TMDs may act both as passive anchors pulled by SNAREpins to bring membranes into close apposition^27^,^28^ and as active components of the fusion machinery, perturbing membrane integrity as they zipper^20^,^25^,^26^,^50^. Quite unexpectedly, we found fusion pore lifetimes were dramatically prolonged in the presence of modifications of the VAMP2-TMD designed to perturb putative interactions with the t-SNARE TMD, in particular for the v3x mutant (Fig. 5). To prevent pore resealing, the TMD mutants must linger at the pore site during most of the pore lifetime. In order to linger at the pore site for up to a minute despite a large entropic drive to disperse into the plasma membrane, the TMD mutants must have favourable interactions with the pore. We suggest the basis of these interactions are geometric and that the TMD-zipper mutant complexes linger at the pore because their geometry and the pore shape fit well together.

It is possible that our TMD manipulations affected fusion rates and pore lifetimes not through disruption of putative v- and t-SNARE TMD interactions, but rather by changing VAMP2 TMD-lipid interactions. Although we cannot exclude a contribution from modified TMD-lipid interactions, we think it is unlikely they were the main driver for the observed phenotypes, for the following reasons. First, v3x produced the largest effect on fusion rates and pore lifetimes, even though it introduced the most subtle modification of the TMD sequence as far as TMD-lipid interactions are concerned (replacing 3 isoleucines with alanines). In contrast, lipid-anchored VAMP2 produced the weakest phenotype, yet it completely lacked TMD-lipid interactions. Finally, three independent TMD modifications designed to disrupt putative v- and t-SNARE TMD interactions all resulted in the same qualitative phenotype: lower pore nucleation rates and longer pore lifetimes.

If we accept for the moment that the contacts seen between the v- and t-SNARE TMDs in a recent crystal structure^25^ are due to attractive interactions between those residues, disrupting those interactions should tend the TMDs to veer away from each other in order to explore a larger conformational space, producing an optimal, or spontaneous geometry that would be on average Y-shaped, if no other constraints applied. In practice, of course splaying of the TMDs would only be possible if the membrane geometry allowed it, by having a high curvature such as at the fusion pore (since the TMDs must still reside within the bilayers). If such a Y-shaped post-fusion complex vacated the fusion pore site and were placed into the flat plasma membrane, the TMDs would be forced to come together, because the TMDs of neuronal/exocytotic SNAREs are somewhat shorter than the bilayer thickness^25^,^51^,^52^. In analogy with how differently shaped lipids partition into bilayer^41^,^42^ or fusion pores^53^, we suggest the spontaneous geometry of the post-fusion SNARE complexes determines how they partition between the fusion pore and the flat plasma membrane. Post-fusion complexes formed by wild-type SNAREs are rod shaped, whereas we expect the spontaneous geometry of TMD-zipper mutants to be Y-shaped, as explained above. It is reasonable that the latter would partition more favourably into the fusion pore than the former.

To cause a dramatic prolongation of pore lifetimes, TMD-zipper mutant SNAREs must linger at the pore for much of the pore lifetime. What about the pore residence time of wild type post-fusion SNARE complexes? We can imagine two possibilities. First, these complexes could vacate the pore rapidly (residence time ≪ pore lifetime) and leave the pore lifetime to be determined by lipid bilayer properties. Second, pores could reseal as soon as the rod-shaped wild-type post-fusion complexes left the pore (residence time 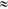 pore lifetime). Interestingly, although rare, once successfully nucleated, fusion pores between SNARE-free NDs and tCells had properties indistinguishable from fusion pores between wild-type vNDs and tCells (Supplementary Fig. 5). This observation seems to favour the first possibility. However, other explanations for the apparent similarity between vND, eND and v4xND pores cannot be excluded. First, because of the extremely low nucleation rate observed with eNDs, the sample size was very low (<20 pores). A substantially larger sample size might reveal some differences. Second, there could be differences that cannot be measured by our method, as different pore geometries can result in the same conductance. For example, a long cylinder with a large radius can produce the same conductance as a shorter but narrower one. Third, it may be that wild-type SNAREs do determine pore properties, but that coincidentally pore lifetimes in the presence of eNDs and vNDs were similar under the conditions used. Using different lipid compositions or different, non-neuronal cognate SNARE pairs may resolve this issue in the future. Finally, during exocytosis a number of other proteins are closely associated with the fusion pore and affect its properties, so one should be careful about generalizing results obtained with SNAREs alone.

The modifications we used to disrupt v- and t-SNARE TMD interactions resulted in stabilized pores, yet the same modifications reduced pore nucleation rates. This apparent contradiction can be resolved if we consider that pore nucleation comprises distinct stages which may be affected differently by the v- and t-SNARE TMD interactions, and that none of the possible pre-fusion configurations are visible to our conductance measurements. We note that there is at least one precedent where replacing the TMD of a fusogen resulted in reduced fusion pore nucleation rate, but once nucleated, pores were stabilized. Markosyan *et al.*^54^ found that GPI-anchored hemagglutinin (HA) of influenza virus was less efficient than wild-type HA in nucleating pores. Once formed, however, GPI-HA pores failed to expand.

We expect our new single fusion pore assay to be useful in elucidating mechanisms by which SNAREs and other components of the fusion machinery determine fusion pore properties much more clearly than was previously possible. The method, and the principle of control of pore dynamics by fusion protein structural intermediate geometry should also be applicable to fusion reactions catalysed by other proteins, such as those that mediate viral or intracellular fusion for which direct probing using electrophysiological or electrochemical methods is difficult.

## Methods

### Stable flipped t-SNARE and wild-type HeLa cell culture

Generation and culture of HeLa stable cell lines co-expressing flipped t-SNAREs (flipped Syntaxin 1 and flipped SNAP-25) and the fluorescent marker CFP-nls (tCells) has been described earlier^32^,^55^. In this cell line, expression of flipped t-SNARE and the fluorescent protein are under the control of a single tetracycline responsive element, which in presence of doxycycline or tetracycline blocks their expression. tCells and wild type HeLa cells were maintained in DMEM (4500 mg/L glucose, L-glutamine, sodium pyruvate and sodium bicarbonate) and 10% (v/v) fetal calf serum at 37 °C. Before electrophysiological recordings, tCells were cultured at least five days in the absence doxycycline or tetracycline for the full expression of flipped t-SNARE proteins. Cells were passaged every two days by detaching them using a sodium citrate solution (11 g/L KCl, 4.4 g/L Na Citrate). After at most three weeks of cell culture, a new aliquot of cryopreserved HeLa cells was thawed.

### Quantification of flipped t-SNARE cell surface density

Cell surface biotinylation, followed by immunoblotting for SNAP25 was used to quantify the average number of flipped t-SNAREs on the surface of a cell^30^. Together with estimates of total cell and patched membrane areas from capacitance and microscopy measurements, respectively, and assuming flipped t-SNAREs distributed homogeneously over the cell surface, this provided an estimate of the flipped t-SNARE density.

Biotinylation of cell surface proteins was performed as described previously^30^. Briefly, tCells were grown ≥5 days in 10 cm tissue culture dishes. At 80% confluency, cells were lifted off the surface using a sodium citrate solution, then rinsed twice in cold PBS supplemented with 0.1 mM calcium and 100 mM magnesium (PBS++), and resuspended in 2 ml cold PBS++. A 10 μl aliquot was used to count the number of cells, while 2 ml of a biotin solution (EZ-link sulfo–NHS–LC-biotin, Thermo Scientific Pierce Biotech, 1 mg/ml in PBS++) was incubated with the rest of the cells for 30 min at 4 °C with occasional gentle shaking. Cells were then washed twice with PBS++ containing 100 mM glycine and incubated in the same solution for 45 min at 4 °C with gentle agitation to quench any unbound biotin. Cells were lysed and the biotin-labeled surface proteins were pulled down using streptavidin-agarose beads. The amount of flipped t-SNAREs per cell (=14,690 ± 950, mean ± S.E.M., n = 3) was estimated using quantitative Western blotting with an anti-SNAP 25 monoclonal mouse antibody (1:2,000, Synaptic Systems, Goettingen). The average area per cell (908 ± 47 μm^2^, mean ± S.E.M., n = 13 cells) was estimated from whole-cell capacitance measurements (see Electrophysiology below), assuming a specific capacitance of 10 fF/μm^2^. Patch area was estimated from the size of the pipette tip (4.9 ± 0.4 μm^2^, mean ± S.E.M., n = 7). These values imply 16 ± 1.0 t-SNAREs per μm^2^ and 79 ± 5 per patch. This is a lower bound, since the actual patch area is expected to be larger than the pipette opening^56^. Since every vND carries 4–5 vSNAREs per face, fusion of many vNDs with the patch is possible.

### Plasmid constructs and Protein Purification

For expressing the t-SNARE complex used in vND-tSUV fusion experiments, we used a polycistronic vector coding rStx1A (no tag) and His_6_-mSNAP25 (plasmid pTW34). Expression in BL21 (DE3) cells and purification were as described in Parlati *et al.*^57^. Briefly, cultures were grown in LB media at 37 °C to an OD600 of 0.65. The cells were then induced with 1 mM IPTG for 3 hrs at 37 °C. The cells were pelleted and then resuspended in breaking buffer (25 mM Hepes pH 7.4, 400 mM KCl, 10% glycerol, 5% Triton X-100, 1 mM TCEP, protease inhibitor). The cells were lysed in a cell disruptor 3 times and the lysate was cleared with centrifugation at 40,000 rpm for 45 min. The lysate was incubated with nickel-NTA resin overnight at 4°C. The lysate was collected and the resin was washed with 25 mM Hepes pH 7.4, 400 mM KCl, 10% glycerol, 1% OG, 1 mM TCEP, followed by a wash with 25 mM Hepes pH 7.4, 400 mM KCl, 10% glycerol, 1% OG, 1 mM TCEP. The proteins were eluted off the column in 25 mM Hepes pH 7.4, 400 mM KCl, 10% glycerol, 1% OG, 1 mM TCEP, 400 mM imidazole.

For the cytoplasmic domain of VAMP2 (CDV), we used a SUMO-CDV construct. Cells were grown, the protein expressed and purified in the same manner as for the recombinant t-SNAREs above, except cell pellets were resuspended in 25 mM Hepes pH 7.4, 140 mM KCl, 10% glycerol, 1 mM TCEP, and protease inhibitor. The lysate was incubated with nickel-NTA resin for 4 hrs at 4 °C. Lysate was collected and the column was washed with the breaking buffer and then the same buffer with the addition of 50 mM imidazole. The column was capped and 1 mL of the breaking buffer (minus the protease inhibitor) was added to the column along with SUMO protease. Cleavage was allowed to proceed overnight at 4 °C. Cleaved CDV was eluted off the column in breaking buffer (minus the protease inhibitor).

We followed Giraudo *et al.*^31^ for expression and purification of the tetanus neurotoxin light chain TeNT.

The plasmid pET-SUMO-VAMP2 was used to produce full length WT VAMP2 protein^20^. VAMP2-I98A/I102A/I106A was generated with a QuickChange site-directed mutagenesis kit (Stratagene, La Jolla, CA) and standard cloning and ligation techniques were used to generate the chimeric VAMP2-Bet1 in which the natural VAMP2 TMD and lumenal residues (96–116) were replaced with those from Bet1 (residues 96–118). pJM51, a previously described construct^27^, was used to produce VAMP^95^Cys, which contains the entire cytoplasmic domain (residues 1–95) with a C-terminal cysteine residue. MSP1E3D1 expression vector (pET28-MSP1E3D1) was purchased from Addgene Inc.

WT and mutant VAMP2 proteins were expressed and purified as described previously^20^, except the proteins were cleaved off the Ni^2+^-beads by SUMO protease overnight at 4 °C. MSP1E3D1 was expressed and purified as previously described^58^ with MSP proteins cleaved directly off the column by TEV protease overnight at 4 °C. In all cases, protein concentrations were determined by the Bradford Assay (Biorad) using bovine serum albumin as standard. VAMP with a lipid anchor was prepared as described earlier^27^. Briefly, the maleimidopropionic acid solanesyl ester (Maleimide-C45) produced as previously described^20^ was dried under N_2_ gas, further dried under vacuum, then mixed with VAMP^95^-Cys in a buffer composed of 25 mM HEPES, 140 mM KCl, and 1% octylglucoside, pH 7.4. The protein-to-lipid ratio was 1:10 to allow for complete coupling. The mixture was vortexed for 30 min at room temperature and the coupling reaction was allowed to proceed for 1h without shaking. The sample was purified using Ni-NTA beads to remove unreacted C45 lipid.

### Nanodisc preparation

Nanodiscs were prepared as previously described^20^,^59^. Briefly, a lipid mixture of palmitoyl-2-oleoyl phosphatidylcholine (POPC) and 1,2-dioleoyl phosphatidylserine (DOPS) at 85:15 molar ratio (for content release) or POPC:DOPS: N-(7-nitro 2,1,3- benzoxadiazole-4-yl)-1,2-dipalmitoyl phosphatidylethanolamine (NBD-PE): N-(Lissamine rhodamine B sulfonyl)-1,2-dipalmitoyl phosphatidylethanolamine (LR-PE) at 82:15:1.5:1.5 molar ratio (for fluorescence measurements) were dried under nitrogen flow for 15 min, followed by vacuum for 1 h. The lipids were re-suspended in 25 mM HEPES, 140 mM KCl, pH 7.4 buffer with MSP and the VAMP2 (WT or mutant) protein containing 1% octylglucoside by rapid mixing for 15 min. Typically, the molar ratios were MSP:SNAREs:lipid = 1:4:60. The samples were then incubated for 3 h at 4 °C with mild shaking. Following this, SM-2 Biobeads (BioRad) were added to the samples and incubated overnight at 4 °C to remove excess detergent. Assembled nanodiscs were separated from un-incorporated proteins and empty nanodiscs by gel filtration on a Superdex 200 column (GE Healthcare). The samples were concentrated and analyzed by SDS PAGE-Coomassie Stain. The number of copies of VAMP WT or mutant in the disc were determined by the VAMP/MSP ratio (assuming 2 MSP protein per nanodisc) according to the quantification of the protein bands on the gel. All NDs were loaded with 7–9 total copies of VAMP2, the maximum that these NDs can bear^20^, unless indicated otherwise.

### Liposome Preparation and Content release assay

t-SNARE containing liposomes loaded with calcium were prepared and release was assayed as described previously^59^.

### Single-cell lipid mixing assay

For monitoring lipid mixing as a function of time, tCells were plated in 35 mm poly-D-lysine coated glass bottom dishes (MatTek Corporation, MA, USA) and incubated in DMEM supplemented by 10% (v/v) foetal calf serum at 37 °C until 80–85% confluence was reached. Before the assay, tCells were washed 3 times with ice cold PBS–, then 100 μl NDs (15 μl ND stock diluted in ice-cold 85 μl PBS– buffer to final lipid concentration of 36 μM) were added to a total volume of 1 ml by PBS—buffer covering the cells. ND lipids included 1 mole % each of DiI and DiD fluorescent lipid labels. tCells were incubated at 4 °C for 30 min, a temperature at which trans-SNARE complexes assemble, but are unable to induce fusion^15^. tCells were then washed twice with ice cold PBS–. The dish was mounted onto the microscope stage (37 °C, 5% CO2), 1 ml PBS– at 37 °C was added and imaging started after focusing. Images were acquired through a Nikon Plan Apo λ 60×/1.45 oil immersion lens using an Andor DU-888 EMCCD camera (Andor Technology Ltd., Belfast, 1024 × 1024 pixels full frame, 3.077 pixels per micron) coupled to a Yokogawa CSU-W1 spinning disk confocal head. Fluorescence time-lapse image cycles were acquired every 30 s for 20 min. Each image cycle consisted of rapid sequential imaging of DiI and DiD channels, excited at 561 nm and 647 nm respectively. Once every 10 cycles we also imaged CFP fluorescence (which labelled nuclei), excited at 488 nm.

We used a slightly different protocol to assess lipid mixing at a single time point that avoided the cold incubation step of NDs and tCells. tCells were cultured as above on glass bottom dishes. A dish was mounted onto the microscope chamber held at 37 °C and supplied with 5% CO2. A 100 μl ND solution as above was added into the dish. After 15 min incubation, cells were washed twice with PBS—buffer and imaged using the same microscope as above. Time-lapse imaging was not possible during the incubation period due to the high background from excess NDs. Fluorescence of DiI, DiD and CFP were excited at 561 nm, 647 nm and 488 nm respectively. We acquired z-stacks by shifting the focus in steps of 1 μm and retained frames that were focused within cells.

#### Image analysis

We used Matlab Image Analysis Toolbox to detect cell contours in the DiD channel using a Canny edge detection algorithm. We created a mask by dilating the detected edges by 5 pixels and calculated pixel statistics corresponding to the mask in both the DiI and DiD channels. We calculated the DiI to DiD ratio image through pixel-by-pixel division of pixel values (converted to double precision) and extracted the mean ratio for each image cycle. We visually checked that edges were correctly detected by the algorithm for every DiD image.

### Single-cell calcium influx assay

tCells seeded in 35 mm poly-D-lysine coated MatTek glass bottom dishes were cultured in DMEM supplemented by 10% (v/v) foetal calf serum at 37 °C until 80–85% confluence was reached. tCells were washed three times with PBS– before being exposed to 5 μM of cell permeable acetoxymethyl ester (AM) conjugated calcium-sensitive fluorophore Fluo-4 (life technologies, NYC, USA) for 30 min at room temperature. After Fluo-4 AM loading, tCells were washed twice with PBS– and incubated in the same buffer for an additional 10 min for recovery before addition of 900 μl PBS++ (containing 1 mM Mg^2+^; 2 mM Ca^2+^). Cells were then mounted on the stage of a Nikon Eclipse Ti confocal microscope held at 37 °C. Imaging of Fluo-4 fluorescence (excited at 488 nm) started immediately after addition of 100 μl NDs solution (15 μl of ND stock solution dissolved in 85 μl PBS++, final lipid concentration 36 μM). We used a Plan Fluor 40×/1.30 oil immersion lens using an Andor DU-888 EMCCD camera coupled to a Yokogawa CSU-W1 spinning disk confocal head. Time-lapse images of Fluo-4 signals were recorded every 5 s for 20 min. We calculated changes in fluorescence intensity relative to the initial value, ΔF/F_0_ as a function of time (Supplementary Fig. 8).

### Influx of fluorescent false neurotransmitters (FFNs) into tCells

Flipped t-SNARE HeLa cells (tCells) were plated in 35 mm poly-d-lysine coated glass bottom dishes (MatTek Corporation, MA) and incubated in DMEM (4500 mg/L glucose, L-glutamine, sodium pyruvate and sodium bicarbonate) and 10% (v/v) foetal calf serum at 37 °C until a confluence of 80–85% was achieved. Prior to experiments, tCells were washed three times with PBS buffer (Life Technnologies, NYC, USA) and then were mounted on the stage of a Nikon Eclipse Ti microscope in a recording chamber (37 °C with 5% CO2). Background fluorescence images from ten randomly selected locations on the dish were captured using a Nikon CFI Plan Apochromat Lambda 60×/1.4 oil immersion lens and an Andor iXon Ultra888 EM-CCD camera (Andor Technology Ltd., Belfast, UK). Afterwards tCells were exposed to 200 μM of FFN202 (ab120867; Abcam, Cambridge, UK) in PBS loading solution (with or without 15 μl NDs to a final concentration of ~90 nM NDs, 36 μM lipids) for 20 min in the recording chamber. After FFN202 loading, tCells were washed 3 times by PBS buffer, 1 ml PBS buffer was added to the dish, and measurement of FFN202 signals was immediately started. Ten images were captured from randomly selected areas on the dish. FFN202 fluorescence was excited at 390 nm and collected through standard DAPI emission filters. Background-corrected FFN202 fluorescence, ΔF, was plotted for the different conditions in Supplementary Fig. 9.

### Proteolysis of v-SNAREs by clostridial neurotoxin light chains

tCells were seeded in Corning TC-treated culture dishes (80.5 mm diameter by 20 mm height, Corning Life Sciences, NY, USA) and incubated in DMEM supplemented by 10% (v/v) foetal calf serum at 37 °C until 80% confluence. For toxin cleavage experiments, tCells were washed three times with PBS– (lacking Mg^2+^ or Ca^2+^, pH 7.2, Life Technnologies, New York) buffer and then were exposed to ~90 nM of nanodiscs (36 μM lipid) for 30 min at 37 °C. vNDs, vNDs pretreated by TeNT (at least 30 min at 37 °C) or v4xNDs in PBS– buffer were used. tCells were rinsed three times by PBS– buffer. TeNT or BoNT/B was added and the cells were incubated a further 30 min at 37 °C. After toxin treatment, cells were washed three times with ice-cold PBS+ (PBS supplemented by 0.1 mM Ca^2+^ and 1 mM Mg^2+^). A 10 μl aliquot was removed to count the number of cells, while 2 ml of a biotin solution (EZ-link sulfo–NHS–LC-biotin, Thermo Scientific Pierce Biotech, 1 mg/ml in PBS+) was incubated with the rest of the cells for 30 min at 4 °C with occasional gentle shaking. Cells were lysed and the biotin-labelled surface proteins were pulled down using avidin-agarose beads (Thermo Scientific Pierce Biotech, New York). The MSP and VAMP2 proteins on the western blots were detected by the Anti Apo-A1 (Apolipoprotein A1 Antibody; 1:2000; Life technologies) and Anti VAMP2 (VAMP2 Antibody; 1:800; Life technologies). Although we started with about the same number of cells per dish (2.5 × 10^7^) and loaded the gels the same volume for the different conditions, there was some variability in the recovery of proteins. Therefore, we used the sodium-potassium ATPase as a plasma membrane protein loading control (detected using an anti-sodium potassium ATPase antibody, Abcam, MA, USA). To analyse Western Blots, we set the band intensities for VAMP2 or VAMP2-4X to 100% after normalizing each band with respect to the intensity of the loading control band. We repeated the experiments three times.

### Electrophysiology

Flipped t-SNARE HeLa cells (tCells) grown in a 3 cm culture dish were placed onto the temperature-controlled stage (TC-202A by Harvard Apparatus, or Thermo Plate by Tokai Hit) of an inverted microscope (Olympus IX71, Olympus Corp.) equipped with an EMCCD camera (DU-885K, Andor) controlled by Solis software (Andor). Recording pipettes (borosilicate glass, BF 150-86-10, Sutter Instruments) were pulled using a model P-1000 pipette puller (Sutter Instruments) and polished using a micro-forge (MF-830, Narishige). Pipette resistances were 5–10 MΩ in NaCl-based solution. The bathing medium was composed of (in mM): 125 NaCl, 4 KCl, 2 CaCl_2_ 1 MgCl_2_, 10 HEPES, (pH adjusted to 7.2 using NaOH) for the cell-attached or whole-cell recordings. The medium was supplemented with 10 mM glucose before use. All voltage-clamp recordings were made using a HEKA EPC10 Double USB amplifier (HEKA Electronics, Inc.), controlled by the Patchmaster software (HEKA). Currents were recorded using 5–20 mV/pA gain, and a sampling rate of 10 or 20 kHz and filtered at 3 kHz. All experiments were done at 35–37 °C. Cells were kept <2 hours on the microscope stage during recordings.

To measure SNARE-mediated single fusion pore currents in the cell-attached mode^60^, electrodes were filled with a solution containing (in mM) 125 NaCl, 4 KCl, 1 MgCl_2_, 10 HEPES, 13 or 26 tetraethylammonium-Cl (TEA-Cl, K^+^-channel antagonist), adjusted to pH 7.2 using NaOH. The resistivity of this solution was 0.60 Ohm.m, measured using a conductivity cell (Orion Versa Star, Thermo Scientific). The pipette tip was initially filled with 1 μl of ND-free buffer and back-filled with vNDs suspended in the same buffer (final [vND] 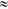     150 nM, or 60 μM lipid). This allowed establishing a tight seal (*R*_*seal*_ > 10 GOhm) with high success rate and recording a stable baseline before the vNDs diffused to the membrane patch and started fusing with it. A similar back-filling strategy is commonly employed in perforated patch measurements^33^. All cell-attached recordings were performed using a holding potential of −40 mV relative to bath, except for experiments that probed the initial pore expansion at high time resolution, wherein the pipette potential was set to 0 mV. With a cell resting membrane potential of −56 ± 7 mV (mean ± S.D., n = 36), this provided 16 mV driving force across the patch membrane (or 56 mV for the high time resolution recordings). For experiments to confirm the small size of pores, electrodes were filled with a solution containing (in mM): 129 N-methyl-d-glucamine (NMDG^+^), 10 HEPES, 26 TEA-Cl, pH adjusted to 7.2 using HCl, resistivity 0.88 Ohm.m.

To quantify diffusion of NDs to the pipette tip (Supplementary Fig. 1), we filled the pipettes as above, but used NDs labelled with the lipid probe LR-PE. We used time-lapse fluorescence imaging, with focus set at the pipette tip (exposed to air to avoid flows due to slight pressure differences inside and outside the pipette were the tip immersed in solution), using a Prior Lumen200Pro illuminator, a Semrock Brightline filter set (TxRed-4040C), and an Olympus LUCPlanFL 40×/0.60 objective.

To measure whole-cell currents elicited upon application of NDs, pipettes were filled with intracellular solution (in mM): 119 KCl, 2 MgCl2, 10 HEPES, 5 EGTA, 26 TEA-Cl (pH is adjusted to 7.2), and a cell voltage-clamped at −70 mV. A second pipette (diameter 2.5–3 μm) filled with vNDs (0.5 μM in the same pipette solution used for on-cell recordings) was placed nearby. NDs were puffed using a pressure-driven dispenser (Picospritzer III, Parker Instrumentation). Whole cell currents were digitized at 20 kHz and filtered at 3 kHz using a HEKA EPC10 amplifier.

### Activity of stretch-sensitive channels

To vary the patch membrane tension, we controlled the pressure above the pipette solution using a high-speed pressure clamp device^61^ (HSPC-1, ALA Scientific Instruments, Westbury, NY). The device can generate reproducible and rapid pressures (both negative and positive) within milliseconds. HSPC-1 was controlled via analogue signals sent from the HEKA EPC10 Double USB amplifier. Pressure values were also monitored using the analogue-to-digital input of the EPC10, according to the calibration values provided by the manufacturer of the HSPC-1. A cell was patched in the cell-attached configuration. While the pipette was held at −40 mV, current was monitored at a given pressure for a period of 10 or 40 s. Pressure was increased in increments of 10 mm Hg from 0 mm Hg to 100 mm Hg and the current measurements repeated. All experiments were performed at 37 °C. The open probability (P_o_) of single channels (currents larger than or equal to1 pA and opening longer than 10 ms) was calculated by dividing the total time spent in the open state by the total time of continuous recording (10 s or 40 s) in stretches of data containing due to a single active channel. We used the same solutions as for fusion pore recordings except for Mg^2+^ or Gd^3+^, which were adjusted as shown in Supplementary Fig. 6, and inclusion of 1 μM Ca^2+^-channel blocker Efonidipine (E0159, Sigma).

### Data analysis

Patchmaster (HEKA Electronik) current traces were exported to Matlab (Mathworks). An interactive graphical user interface was designed in Matlab to identify, crop, and process single fusion pore currents. Traces were low-pass filtered using a pass-band of 280 Hz (2.8 kHz for high time resolution recordings), and sharp frequency peaks due to digital noise and/or line voltage identified from the baseline were removed using notch filtering, using zero-phase digital filtering to avoid signal distortion. Filtered traces were averaged in blocks of 40 or 80 points to achieve 4 ms between points (125 Hz bandwidth), with rms baseline noise ~0.2 pA (span 0.06–0.7 pA). Traces with larger than 0.7 pA noise were rejected. For high time resolution recordings, blocks of 8 successive points were averaged, such that in the final traces points were spaced by 0.4 ms each (1.25 kHz bandwidth). Baseline noise was ~0.6 pA (span 0.30–0.89) and traces with noise >1 pA were rejected. After baseline correction, currents 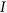 for which |*I*| > 2.0 pA for at least 250 ms were accepted as fusion pore current bursts. A burst included rapidly fluctuating currents that briefly returned to baseline. To quantify pore flickering, we defined currents <−0.25 pA and lasting ≥60 ms (15 points) as open pores and currents not meeting these criteria as closed. For high time resolution recordings, we set −0.875 pA and a minimum crossing of 15 points (6 ms) as the threshold.

Our pore detection and analysis criteria necessarily deviate from standard single ion-channel analysis techniques, because the fusion pores we measure do not have well-defined, discrete conductance states. In single ion channel analysis, it is typical to set a pore detection threshold equal to 3–5 times the rms baseline noise, or half the single-channel conductance. The latter approach could not be applied here in the absence of well-defined conductance states, while the former approach would eliminate all pore conductance values below a large cut-off that depended on acquisition conditions, not physical considerations. This would bias pore conductance and radii estimates toward larger values. Instead, we chose a threshold that was slightly larger (in absolute value) than the root mean squared (rms) noise that reflected the smallest pores that could meaningfully be measured (~0.2 nm, the size of a hydrated Na^+^ ion, corresponding to ~16 pS assuming a 15 nm long cylindrical pore). This of course made it likely that a purely random current excursion cross the threshold. To reduce such false-detections, we additionally imposed that a minimum number of successive data points cross the threshold to be considered an open pore. If we knew the distribution of baseline current values around the mean (noise distribution), and if successive points were uncorrelated, then we could calculate the probability, *p*_*o*_, of a single or *N* successive threshold crossings, and consequently estimate the false-detection rate. In general the noise distribution is difficult to estimate a priori, and successive points may be highly correlated, since various sources of noise and filtering contribute. However, if points are averaged in blocks of sufficiently large size, the Central Limit theorem ensures that noise in the block-averaged trace is normally distributed and successive points in the noise trace are uncorrelated. In addition, block averaging reduces the number of data points and makes computations and data handling easier. For these reasons, block averaging is commonly used, e.g. in analysis of optical tweezer data^24^,^62^. We thus averaged the data in blocks of 40 or 80 to increase the spacing between successive points to 4 ms. Using block-averaged traces, we plotted the noise distribution calculated from the baseline preceding each event, fitted it with a Gaussian, calculated the standard deviation, and checked that the fit was satisfactory. With an rms noise *σ* = 0.2 pA and a threshold *x* = −0.25 pA, the probability *p*_*o*_ of a random current excursion below the threshold is calculated from the cumulative distribution function 
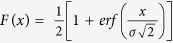
 to be *p*_*o*_ = 0.106. The probability that *N* = *15* successive points are below the threshold is (*p*_o_)^*N*^ = 2.3 × 10^−15^. Thus, with these criteria, false detection in a typical recording lasting 800 s, or 2 × 10^5^ points is negligible. Similar considerations applied to our selection of pore detection criteria for high time-resolution recordings.

The greatest reduction in our temporal resolution occurs during the low-pass filtering stage, which reduces the useful bandwidth of the signal roughly to the filter cutoff frequency *f*_*c*_. Even though the data points are still spaced at 1/(sampling rate), they are only useful for detecting changes in signal that occur in time period 1/*f*_*c*_ or slower. Thus, averaging in blocks does not result in any loss of information, as long as the period is ≤1/(2*f*_*c*_) (the factor of 2 is due to the Nyquist theorem: to reconstruct a signal with frequency *f*, we need to sample at frequency 2*f*), but is advantageous, as explained above.

Examples of fusion pore bursts and detected open periods are shown in Fig. 1d and Supplementary Fig. 2. For a given burst, the number of open periods was equal to the number of flickers. Some recordings ended with what seemed to be currents from overlapping fusion pores. However, since a loose seal could not easily be discriminated from overlapping fusion pores, we did not analyse such end-of-record currents. For vND-tCell fusion for which there were >a few fusion pores per patch, this resulted in an underestimation of the fusion pore nucleation rate. Periods of recordings during which the baseline was unstable (typically the first few minutes) were excluded from nucleation rate calculations.

For the distributions of open-pore conductances and radii (Fig. 1g,h), and the durations of open and closed periods (Supplementary Fig. 3), we first computed the probability density functions (PDFs) for individual fusion pores, then averaged the PDFs, to give equal weight to all fusion pores in the averaged PDF. All distribution fits used maximum likelihood estimation. For aligning conductance traces, we shifted the time axis such that t = 0 corresponded to the first data point in a burst. Matlab Curve Fitting Tool was used to fit double exponentials to the averaged conductance traces in Supplementary Fig. 13. To estimate fusion pore size from conductance, we approximated the pore as a cylinder and used the relation^39^ 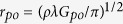, where *ρ* is the resistivity of the solution, *λ* = 15 nm is the length of the cylinder, and 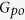 is the open-pore conductance. For assessing statistically significant differences in pairwise comparisons of sample means we used either the two-sample t-test, or the nonparametric two-sample Kolmogorov-Smirnov test (ttest2 or kstest2, Matlab Statistics Toolbox), as indicated in figure legends.

## Additional Information

**How to cite this article**: Wu, Z. *et al.* Nanodisc-cell fusion: control of fusion pore nucleation and lifetimes by SNARE protein transmembrane domains. *Sci. Rep.* **6**, 27287; doi: 10.1038/srep27287 (2016).

## Supplementary Material

Supplementary Information
