# Supplementary material for: Nanodisc-cell fusion: control of fusion pore nucleation and lifetimes by SNARE protein transmembrane domains
Source: Sci Rep. 2016 Jun 6;6:27287. doi: 10.1038/srep27287 (PMC4893671; doi:10.1038/srep27287)
Supplement: Supplementary Information [file srep27287-s1.pdf]

SUPPLEMENTARY INFORMATION

Nanodisc-cell fusion: control of fusion pore nucleation and lifetimes by SNARE protein transmembrane domains

Zhenyong Wu[1,2], Sarah M. Auclair[2,3], Oscar Bello[2,3], Wensi Vennekate[1,2], Natasha Dudzinski[1,2], Shyam S. Krishnakumar[2,3], and Erdem Karatekin[1,2,4,5]*

[1]Department of Cellular and Molecular Physiology, School of Medicine, Yale University, 333 Cedar Street, New Haven, CT, 06520.

[2]Nanobiology Institute, Yale University, 850 West Campus Drive, West Haven, CT, 06516.

[3]Department of Cell Biology, School of Medicine, Yale University, 333 Cedar Street, New Haven, CT, 06520.

[4]Department of Molecular Biophysics and Biochemistry, Yale University, 260 Whitney Avenue, New Haven, CT 06520.

[5]Laboratoire de Neurophotonique, Université Paris Descartes, Faculté des Sciences Fondamentales et Biomédicales, Centre National de la Recherche Scientifique (CNRS) UMR8250, 45, rue des Saints Pères, 75270 Paris Cedex 06, France

*Correspondence to: erdem.karatekin@yale.edu.

SUPPLEMENTARY NOTES

DOCKING AND FUSION OF vNDs WITH tCELLs ARE SNARE-MEDIATED

Our results indicated docking and fusion of vNDs to tCells are SNARE-dependent. We further confirmed this by a toxin cleavage assay. We first incubated vNDs with tCells for 30 min at 37°C, rinsed the cells, tagged cell surface proteins by biotin, pulled them down, subjected them to SDS PAGE, and immunoblotted for VAMP2 or MSP (Supplementary Fig. 10a-c). Treating the vNDs with tetanus neurotoxin (TeNT, cleaves[1] VAMP2) before incubation with tCells resulted in a significant reduction in the amount of both VAMP2 and MSP that was detected (Supplementary Fig. 10a-c).

We next tested whether docking of NDs was mediated by trans-SNARE interactions and if some of the vSNAREs integrated into the plasma membrane after fusion as expected. For this we relied on a differential VAMP2 cleavage assay using TeNT and botulinum neurotoxin serotype B (BoNT/B) light chains. Both toxins cleave VAMP2 at the same site (Q76-F77 bond in the rat or human VAMP2 sequence); however, cleavage requires recognition of different regions of VAMP2 for the two toxins (around 41-45 for TeNT and 63-67 for BoNT/B)[1,2]. If these recognition sites and/or the cleavage site are occluded by zippering into the t-SNARE, VAMP2 is protected from cleavage. Because assembly of v- and t-SNARE complexes proceeds in stages starting from the membrane-distal N-termini[3,4], differential toxin binding can be used to probe the degree of SNARE assembly both *in vivo*[5,6] and *in vitro*[7,8]: VAMP2 in half-zippered SNARE complexes is resistant to cleavage by TeNT, but not by BoNT/B. SNARE complexes zippered beyond roughly the central zeroth layer (R56)[9], either in the pre-fusion *trans* or post-fusion *cis* configuration are protected from either toxin.

We first incubated tCells with NDs for 30 min at 37°C, then rinsed away excess NDs and introduced either TeNT or BoNT/B light chains, or did not introduce any toxin. After a 30 min incubation, we tagged surface proteins with biotin, pulled down and probed biotinylated surface proteins by SDS-PAGE and immunoblotted them for MSP or VAMP2 as above. We reasoned that if NDs loaded with WT VAMP efficiently docked and fused with the plasma membrane, a significant fraction of the VAMP2 should end up either in the fully-zippered pre-fusion (*trans*) or post-fusion (*cis*) configuration, and therefore be protected from proteolysis by either toxin as

schematically shown in Supplementary Figure 10d. Previous work showed that most vNDs remained attached to t-SNARE liposomes after fusion, possibly through a hemi-fission/hemi-fusion structure[10]. Thus, with fusion-competent WT VAMP2 we expected to detect cell surface-associated MSP, though the exact nature of the adhesion is not known. In contrast, the fusion-incompetent VAMP2-4X whose C-terminal half cannot zipper should only bind NDs to the plasma membrane through partially-zippered complexes with t-SNAREs on the cell surface, and be more susceptible to proteolytic attack by BoNT/B than by TeNT (Supplementary Fig. 10). Furthermore, VAMP2-4x cleavage should release docked but unfused NDs (Supplementary Fig. 10d).

As shown in Supplementary Figure 4e,f, our Western blot analyses of biotinylated surface proteins corroborated our expectations. These results are consistent with docking being mediated by v- and t-SNARE interactions in *trans*, followed (in the case of WT VAMP2) by fusion producing toxin-resistant cis-SNARE complexes in the plasma membrane.

COMPARISON OF BULK AND SINGLE-PORE CARGO RELEASE RATES

A previously described bulk fusion assay[10] probes the complex combination of the multi-step kinetics of ND-SUV docking, pore nucleation after docking, and calcium efflux during the lifetime of a pore. Because docking is mediated by the soluble SNARE domains and here we will compare fusion rates only among TMD-modified v-SNAREs, we expect docking rates to be comparable. Thus, any differences in release rates among the TMD-modified SNAREs must arise from differences in the rates of subsequent nucleation and efflux through the fusion pores, provided these rates are comparable to, or slower than, the docking rate.

In bulk calcium release measurements during vND-tSUV fusion, only ~20-30 % of the total entrapped calcium is released during 60 min of reaction (Supplementary Fig. 11a, b). This can be due to two extreme situations: (i) 20-30 % of SUVs each may release its entire contents during a single pore opening (“nucleation-limited release” or “single-shot” release), or (ii) every vesicle may homogenously release 20-30 % of its cargo during 60 min, through multiple pores that open and each release only a small portion of the cargo (“pore-limited release”, or “multiple-shot” release). Nucleation would still contribute to pore-limited release kinetics, but the bulk release rate would be sensitive to average efflux per pore, whereas pore properties would be

invisible for nucleation-limited release. Therefore, comparing bulk cargo release rates among SNAREs whose docking and pore nucleation rates are similar but single-pore efflux rates are different would inform us whether pore-limited release is significant.

In the single-pore experiments, the TMD zipper-disrupted NDs vC45ND, vBet1ND, and v3xND produced similar pore conductances (Supplementary Fig. 12b) and pore open probabilities during a burst (Supplementary Fig. 12a), but very different burst lifetimes (Fig. 5 b,c). Thus, the average number of ions that transfer per pore should scale with the burst lifetimes. In addition, nucleation rates were within a factor of 2-3 for these NDs (Fig. 4b). Bulk calcium release rates for vC45ND-, vBet1ND-, and v3xND-tSUV fusion (Supplementary Fig. 11a, b) correlated with the single-pore burst lifetimes (Figure 5c) suggesting a significant contribution from pore-limited release.

Single fusion pore measurements show that, compared to wild-type v-SNAREs, the TMD zipper-disrupted vND pores nucleate at least 2-5 times slower (Fig. 4b), but last longer (Fig. 5c), allowing more charge to pass per fusion event (Supplementary Fig. 11c). This provides an opportunity to test whether the higher nucleation rate of vND pores can compensate for the longer pore lifetimes of the TMD zipper-deficient SNAREs in the bulk contents release assay. Bulk calcium release rates were comparable for vND- and v3xND-tSUV fusion (Supplementary Fig. 11a, b), suggesting a 10-fold decrease in pore lifetime can be compensated by a corresponding increase in pore nucleation rate.

We extended this analysis by assuming that the relative bulk release rates should reflect the product of nucleation and single-pore efflux rates for pore-limited kinetics. A good measure of the relative rates of cargo efflux through ND-SUV pores is the average charge transferred per fusion pore, q, from single-pore experiments (Supplementary Fig. 11c). When multiplied by the pore nucleation rates measured in the single-pore experiments (Supplementary Fig. 11d), this produced the estimates of relative bulk release rates shown in Supplementary Figure 11e. The estimated rates reflect the actual bulk rates (Supplementary Fig. 11b) rather well, given that we grossly underestimate the nucleation rate for vND-tCell pores because we ignore overlapping or closely spaced current bursts, and that the sensitivity of the bulk assay is lower, unable to distinguish vC45ND rates from background.

In a previous study[10] of bulk fusion rates between vNDs and tSUVs, a lower bound $\gg 100\ \mu s$ for pore lifetimes for calcium release was estimated, but the actual pore lifetime could not be measured. It was also recognized that each pore likely released a fraction of the SUV contents[10]. In light of our vND-tCell single-pore lifetime and size measurements, release of hydrated calcium ions through single vND-tSUV pores may indeed be very slow, lasting seconds, and leading to detectable differences between rapid lipid mixing and slow calcium release signals[10].

REFERENCES:

1 Rummel, A. & Binz, T. *Botulinum neurotoxins*. (Springer, 2013).

2 Sikorra, S., Henke, T., Galli, T. & Binz, T. Substrate recognition mechanism of VAMP/synaptobrevin-cleaving clostridial neurotoxins. *The Journal of biological chemistry* **283**, 21145-21152, doi:10.1074/jbc.M800610200 (2008).

3 Gao, Y. *et al.* Single reconstituted neuronal SNARE complexes zipper in three distinct stages. *Science* **337**, 1340-1343, doi:10.1126/science.1224492 (2012).

4 Sudhof, T. C. & Rothman, J. E. Membrane Fusion: Grappling with SNARE and SM Proteins. *Science* **323**, 474-477 (2009).

5 Hua, S. Y. & Charlton, M. P. Activity-dependent changes in partial VAMP complexes during neurotransmitter release. *Nature neuroscience* **2**, 1078-1083, doi:10.1038/16005 (1999).

6 Prashad, R. C. & Charlton, M. P. SNARE zippering and synaptic strength. *Plos One* **9**, e95130, doi:10.1371/journal.pone.0095130 (2014).

7 Giraudo, C. G., Eng, W. S., Melia, T. J. & Rothman, J. E. A clamping mechanism involved in SNARE-dependent exocytosis. *Science* **313**, 676-680 (2006).

8 Krishnakumar, S. S. *et al.* A conformational switch in complexin is required for synaptotagmin to trigger synaptic fusion. *Nature structural & molecular biology* **18**, 934-940, doi:10.1038/nsmb.2103 (2011).

9 Sutton, R. B., Fasshauer, D., Jahn, R. & Brunger, A. T. Crystal structure of a SNARE complex involved in synaptic exocytosis at 2.4 A resolution. *Nature* **395**, 347-353, doi:10.1038/26412 (1998).

10 Shi, L. *et al.* SNARE Proteins: One to Fuse and Three to Keep the Nascent Fusion Pore Open. *Science* **335**, 1355-1359 (2012).

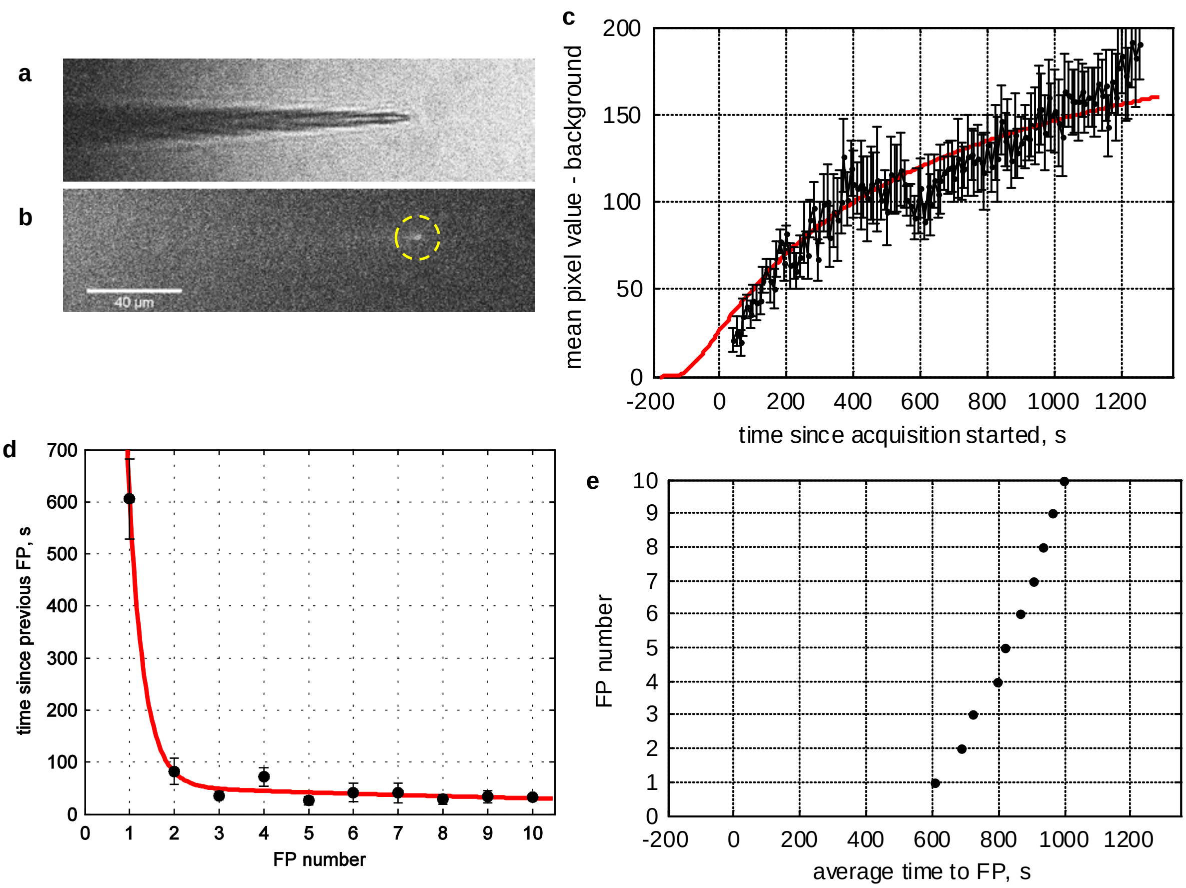

Supplementary Figure 1. Time course of ND diffusion to the pipette tip and pore frequency. **a**. A pipette tip was filled with 1 µl of ND-free pipette solution, then back-filled with fluorescently labelled NDs as in actual vND-tCell fusion pore measurements. Bright field image of the pipette is shown. **b**. The pipette tip (exposed to air) was imaged using fluorescence time-lapse microscopy and the increase in mean pixel value in a circular region of interest (yellow circle) at the pipette tip was measured as a function of time. The left part of the image is brighter due to out-of-focus fluorescence from labelled NDs further away from the tip of the pipette. **c**. Averaged time course from five measurements. If the initially ND-free solution extends a distance l from the tip and the NDs have diffusivity D, we expect the ND concentration at the tip to follow (2) $C = a\left(1 - \text{erf}\left(l/\sqrt{4D(t+t_0)}\right)\right)$, where t_0 is the delay between filling the pipette and the start of the fluorescence time-lapse acquisition (~3 min). Fixing $l = 1$ mm, $D = 10^{-3}$ mm²/s, and $t_0 = 180$ s, and fitting the parameter a, we obtained the solid red curve, with $a = 284$ ($R^2 = 0.89$). **d**. Waiting time distribution for successive fusion pores (FPs). For fusion pore number 1 the delay is from the time patch recordings started. Waiting times between successive fusion pores decrease as the concentration of NDs near the patch increases as a function of time. The red curve is a fit $T = a\exp(-N/N_0) + b\exp(-N/N_1)$, with best fit parameters $N_0 = 0.34$, $N_1 = 16$, $a = 1.0 \times 10^4\ s$, $b = 57$ s, $R^2 = 0.996$. Error bars are standard errors of the mean. **e**. The fusion pore number in a patch as a function of the average time to that fusion pore, measured from the beginning of the recording.

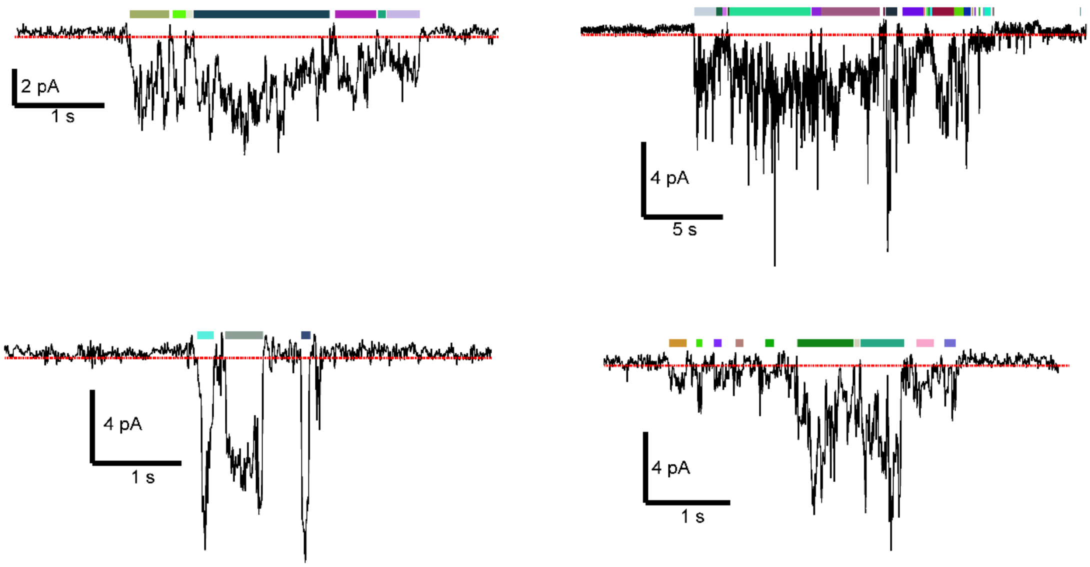

Supplementary Figure 2. Examples of fusion pore current “bursts”. For each trace, the dashed red line is 0.25 pA below the baseline and is the threshold that the trace must cross for at least 60 ms for the pore to be considered open. Open periods detected using this criterion are indicated above the trace as coloured bars.

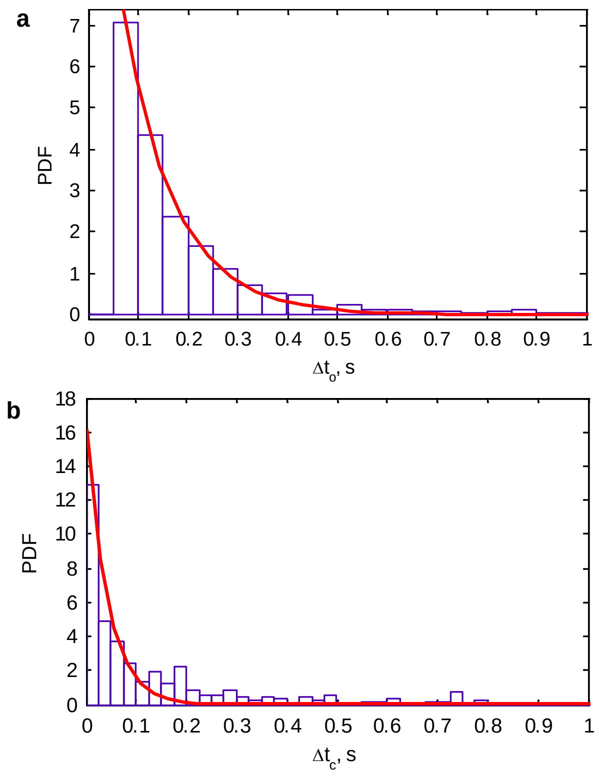

Supplementary Figure 3. Average distributions of open (**a**) and closed (**b**) periods during fusion pore current bursts for vND-tCell fusion (122 individual fusion pores from 50 cells). The red curves are exponential functions, $y = a\ \exp(-t/\tau)$, with the best fit parameters $a = 14.4$ and 16.2 and $\tau = 104$ and 43 ms (R^2 =0.996 and 0.934), respectively for the open and closed periods. Bin widths are 50 and 25 ms for the open and closed period distributions, respectively.

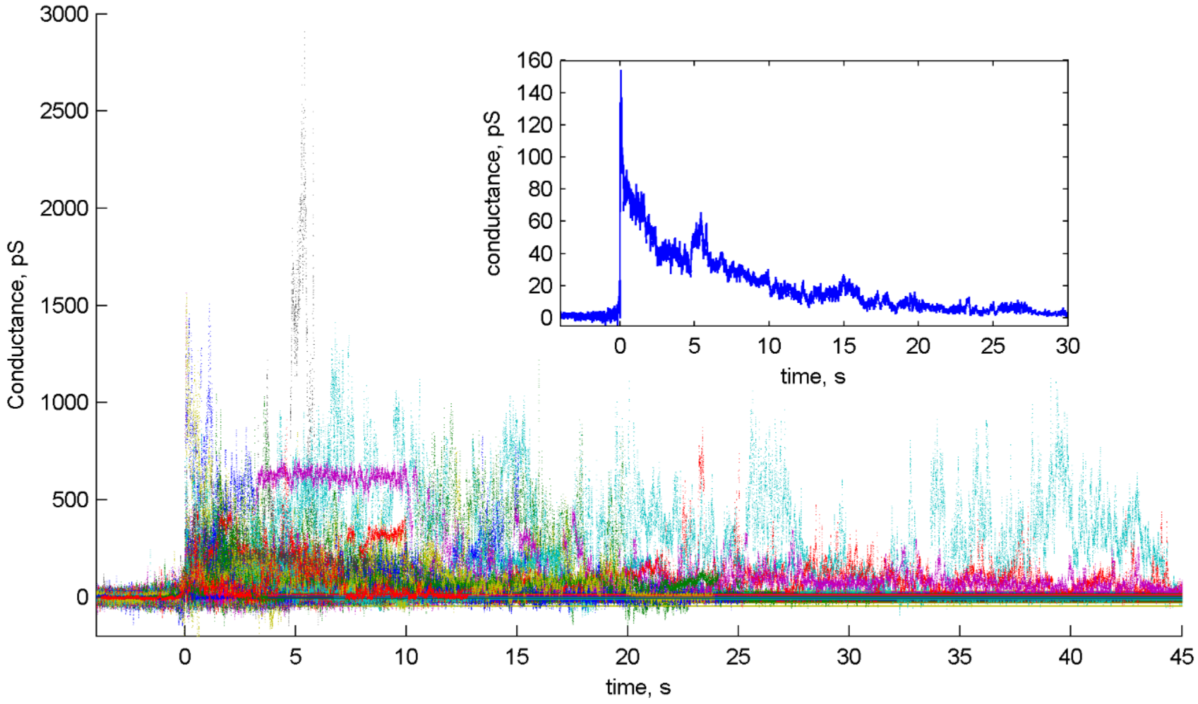

Supplementary Figure 4. Variability in fusion pore conductances. Conductances from 122 vND-tCell fusion pores from 50 cells are plotted together after aligning the time axes to the beginning of the first detected sub-opening in every trace. The average conductance as a function of time is shown in the inset.

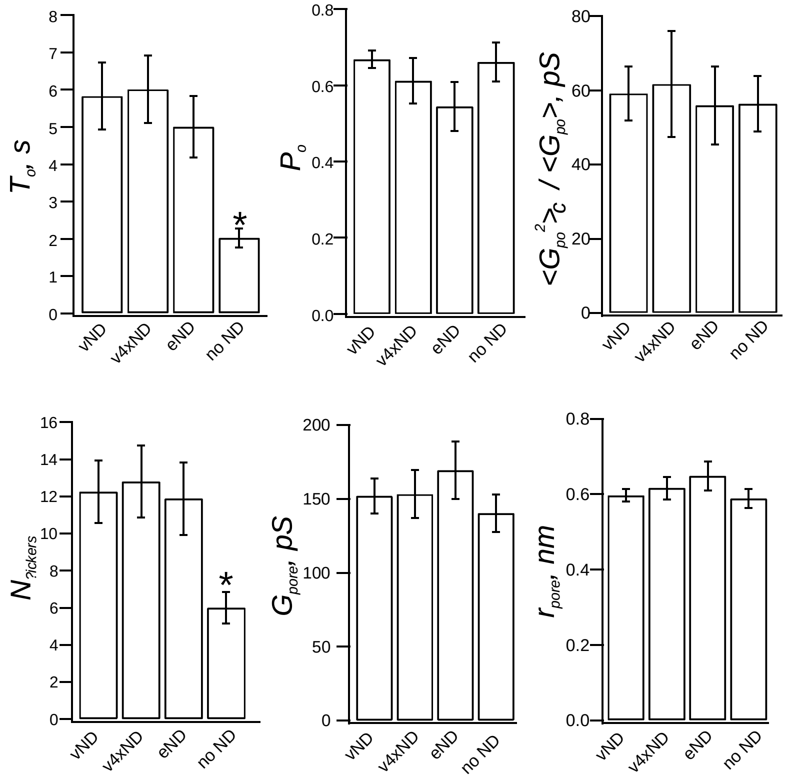

Supplementary Figure 5. Comparison of properties of pore-like events. A flipped t-SNARE cell was patched in the cell-attached configuration. Direct currents under voltage clamp were recorded. Pore-like events were detected and analyzed. Patch pipettes contained no NDs (no ND), SNARE-free NDs (eND), or NDs loaded with WT VAMP2 (vND) or VAMP2-4X (v4xND). The number of patches/pore-like events were 50/125, 29/20, 52/17, 101/31 for vND, v4xND, eND, and no ND conditions, respectively. Statistical significance of differences of all conditions with respect to vND were assessed using the two-sample Kolmogorov-Smirnov test. * indicates $p<0.05$.

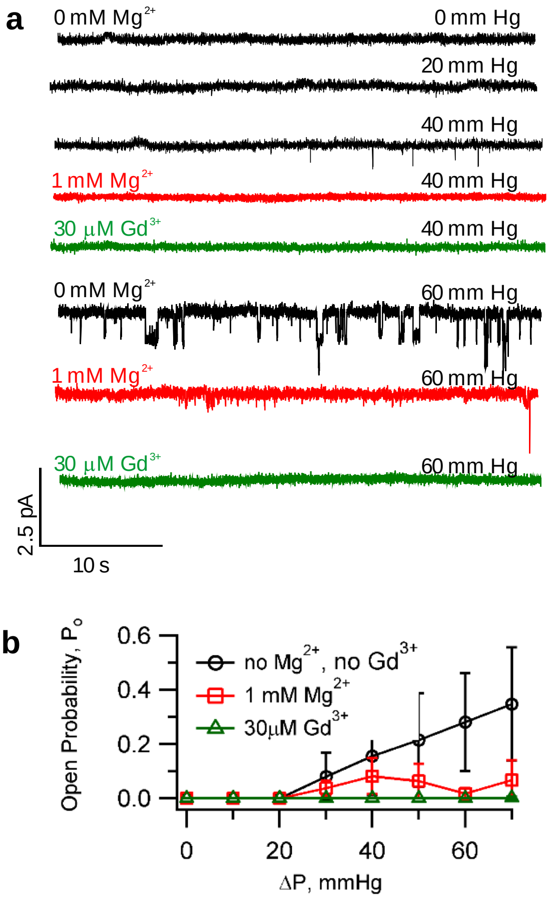

Supplementary Figure 6. Stretch-activated channels do not contribute to fusion pore currents. A flipped t-SNARE cell was patched in the cell-attached configuration and the pipette potential was held at -40 mV. Patch membrane tension was modulated by controlling the pressure inside the pipette using a high-speed pressure clamp device (HSPC-1, ALA Scientific Instruments). Pressure steps were applied from 0 to 100 mmHg in steps of 10 mmHg for 10 or 40 s. **a**. Channel activity was seen only when suction was >20 mmHg. Inclusion of 1 mM Mg^{2+} or 30 µM Gd^{3+} inhibited channel activity. **b**. Channel open probability (fraction of time channels were open during a suction pulse) as a function of suction pressure. The number of patches recorded were 6, 8, and 7 for no Mg^{2+}/Gd^{3+}, 1 mM Mg^{2+} and 30 µM Gd^{3+}, respectively.

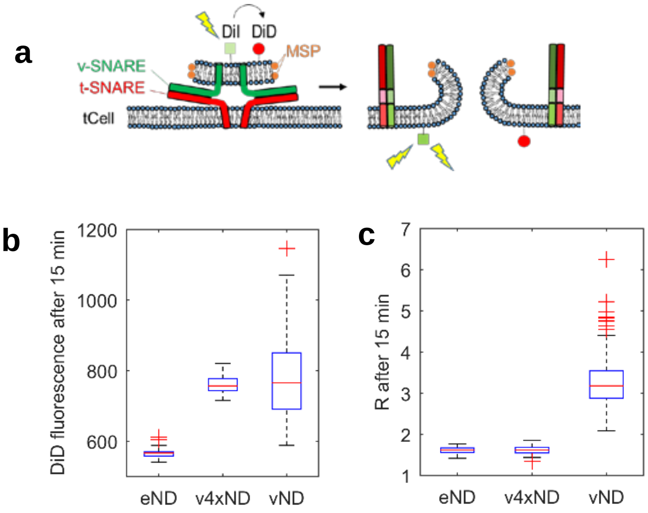

Supplementary Figure 7. Lipid mixing between NDs and tCells. **a**. Schematic of the approach. NDs containing 1% each of DiI and DiD lipid labels were incubated with tCells for 15 min at 37°C, rinsed, then imaged for a single cycle using a spinning disc confocal microscope equipped with a temperature controlled stage set to 37°C. One frame recorded DiI fluorescence excited at 561 nm and the subsequent frame recorded DiD fluorescence excited at 647 nm. DiI fluorescence reports lipid mixing; upon fusion DiI and DiD are diluted in the plasma membrane and DiI is no longer quenched by DiD. At the labelling density used, DiD is not significantly self-quenched, so the DiD signal is proportional to the initial density of docked NDs. The ratio R of DiI-to-DiD fluorescence normalizes fusion signals for variations in docked ND density, temperature-induced fluorescence changes, and other instrumental and environmental factors. **b**. DiD fluorescence shows that v4xNDs docked onto tCells as efficiently as vNDs, but empty NDs were not efficient. **c**. The ratio R that reports lipid mixing is plotted for its value at 15 min. Despite being as effective as wild-type VAMP2 in docking NDs onto tCells, VAMP2-4X is unable to induce lipid mixing. 7, 5, and 7 dishes were used for vND, v4xND, and eND conditions, respectively. For each dish, >70 distinct regions were imaged and analysed.

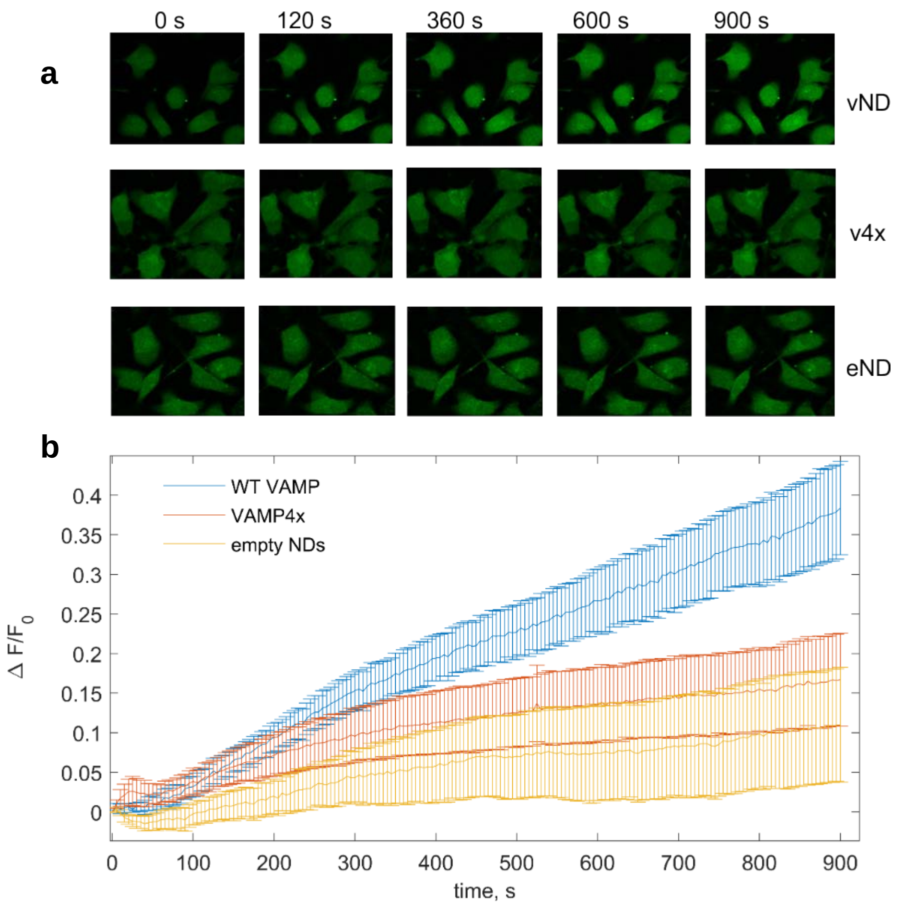

Supplementary Figure 8. Single-cell calcium influx assay. tCells were loaded with the calcium indicator dye Fluo-4. After rinsing away Fluo-4 not taken up by cells, cells were placed onto the stage (held at 37°C) of a confocal microscope. Time-lapse imaging of Fluo-4 fluorescence (excited at 488 nm) started shortly after adding NDs that were empty (eND), loaded with wild-type VAMP2 (vND), or VAMP2-4X (v4xND). NDs did not contain any fluorescent labels. Fusion of NDs with the cell surface would lead to entry of extracellular calcium into the cytosol through fusion pores and result in increased Fluo-4 signals. **a**. Representative snapshots of Fluo-4 fluorescence as a function of time. **b**. Changes in Fluo-4 fluorescence ΔF relative to initial value, F_0. Averages of 5, 8, and 6 acquisitions, each from a separate dish, are shown, for vND, v4xND, and eND samples, respectively. Errobars are S.E.M.

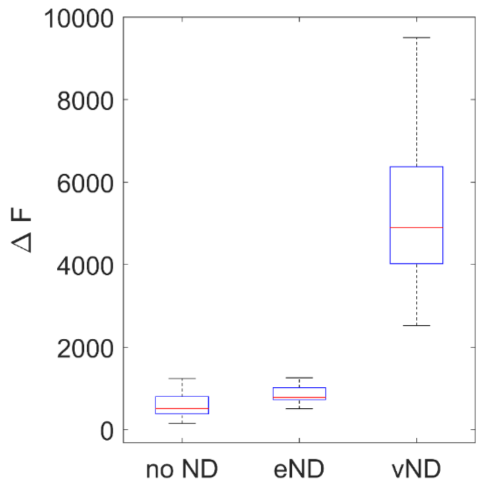

Supplementary Figure 9. Influx of fluorescent false neurotransmitters (FFNs) through fusion pores into tCells. Flipped t-SNARE cells were incubated for 20 min at 37°C with FFN202 in the absence (no ND) or presence of empty NDs (eNDs) or v-SNARE NDs (vND). Cells were were then rinsed and imaged for FFN fluorescence. Background fluorescence (obtained before addition of FFNs) was subtracted from every condition. FFN uptake was higher only under conditions that favour creation of fusion pores, suggesting pores are permeable to FFNs. For every condition two dishes were used and at least 10 distinct areas per dish were imaged and analysed.

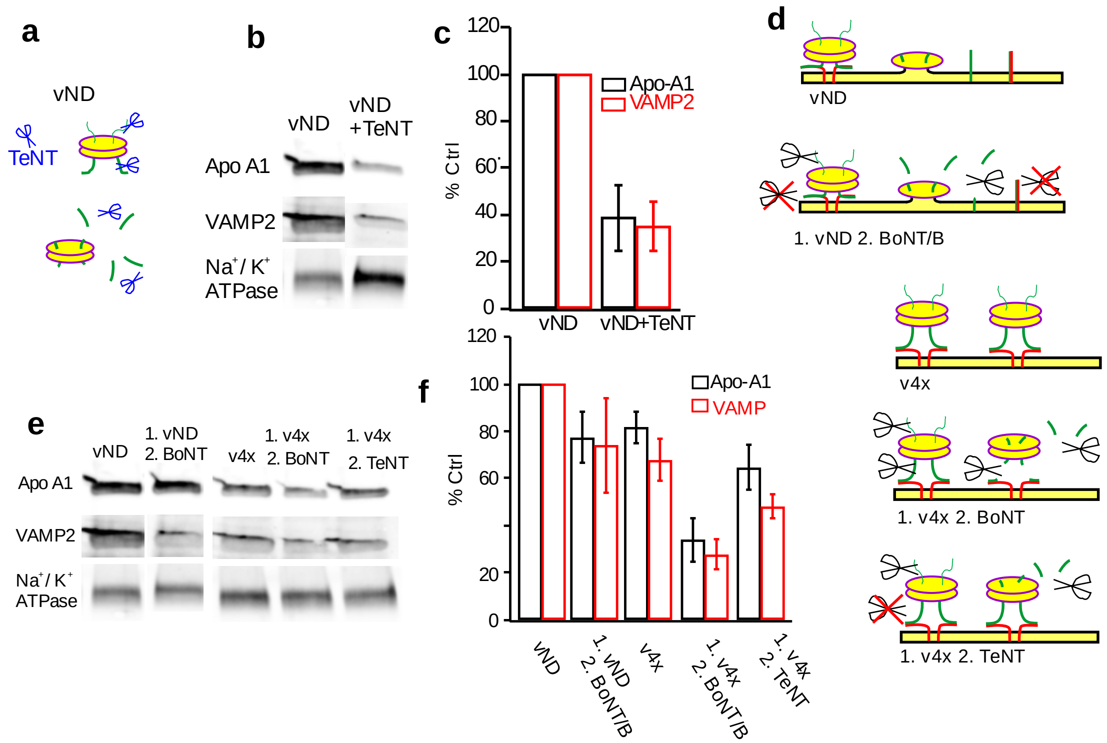

Supplementary Figure 10. Docking and fusion of vNDs with tCells are mediated by SNARE assembly. **a-c**. vNDs were incubated with TeNT for 30 min at 37°C, then incubated another 30 min at 37°C with tCells. tCell surface proteins were biotinylated, pulled-down, and immunoblotted for MSP (using an anti-Apo A1 antibody), VAMP2, or the plasma membrane Na^+/K^+ ATPase (as a loading control). Average of three experiments were quantified (c). A representative blot is shown in b. **d-f**. tCells were incubated with NDs loaded with wild-type (vND) or docking-competent, fusion-incompetent VAMP2-4X (v4x) for 30 min at 37°C. After washing away free NDs, cells were treated with TeNT or BoNT/B for 30 min at 37°C. Cell surface proteins were labelled with biotin, pulled down, and immunoblotted for MSP, VAMP2, or Na^+/K^+ ATPase as before. Expected outcomes are schematically shown in d. Wild-type VAMP2 fully zippered through the SNARE domain either in pre-fusion *trans* or post-fusion *cis* complexes should be protected from proteolytic attack. In contrast, free VAMP2 in ND or cell membranes is prone to proteolysis. VAMP2-4X partially zippers onto the t-SNAREs, occluding the recognition site for TeNT, but not for BoNT/B. Thus, half-zippered VAMP2-4X is prone to proteolytic attact by BoNT/B, but not by TeNT. Western blot analyses (e,f) were consistent with the expected outcomes. Three independent experiments were run and results averaged. Detected levels for vND (both VAMP2 and Apo A1) were set to 100%. e shows a representative blot. Although the same number of cells were treated for each condition and the same volume of recovered sample loaded per well, there was some variability in the amount of recovered material. Therefore, band intensities were first normalized with respect to the Na^+/K^+ ATPase control band for each condition for the quantitative analyses.

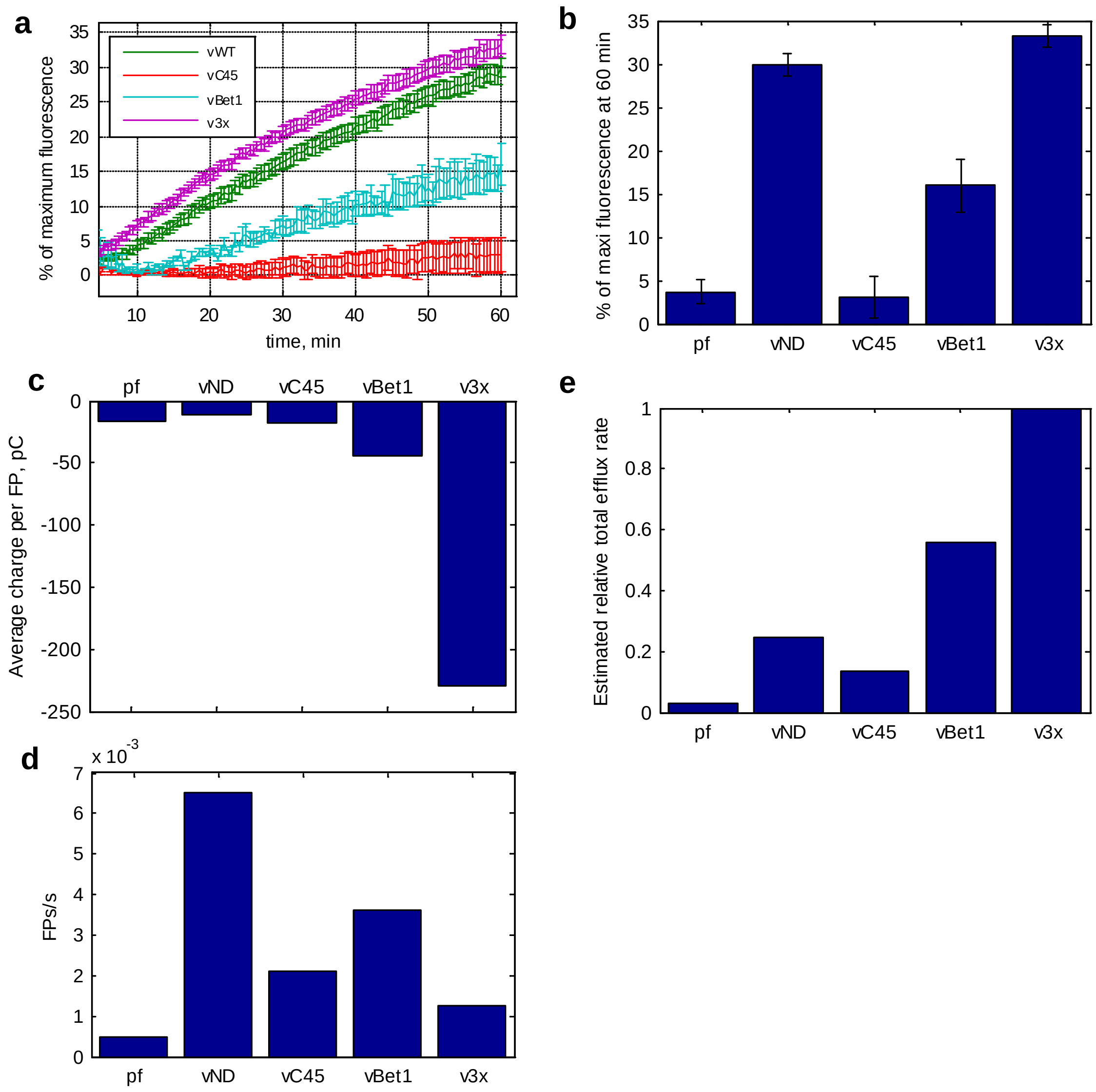

Supplementary Figure 11. Comparison of bulk calcium release during ND-liposome and ND-tCell fusion. **a**. Bulk calcium release during fusion between MSP NDs and t-SNARE reconstituted small unilamellar vesicles (t-SUVs). The MSP NDs contained wild-type (wt) or TMD-modified v-SNAREs as indicated. Initially, 50 mM calcium is trapped inside the t-SUVs. Fusion with a vND results in calcium efflux through the fusion pore. Calcium release is detected by the presence of a calcium-sensitive dye, Mag-Fluo-4 (2 μM, $K_d = 22$ μM), outside the liposomes. Error bars are S.E.M. (n=11, 5, 3, and 8 experiments for vWT, vC45, vBet1, and v3x). **b**. Average calcium released at 60 min as a fraction of the maximum (from a) for the conditions indicated. Also shown are results for SNARE protein-free NDs (pf) **c**. Average charge per fusion pore (FP), q, for the conditions indicated, from single-pore current measurements ($V_m = -16$ mV across the patch membrane). **d**. Single-pore nucleation rates, $\dot{n}$, for the conditions indicated. **e**. Estimate of the relative rates of total release that would be measured in the bulk assay as the product $q \times \dot{n}$, normalized to the largest product (see Supplementary Note for details). pf, vND indicate MSP NDs loaded with 0 (SNARE protein-free), or ~7 copies of vSNAREs.

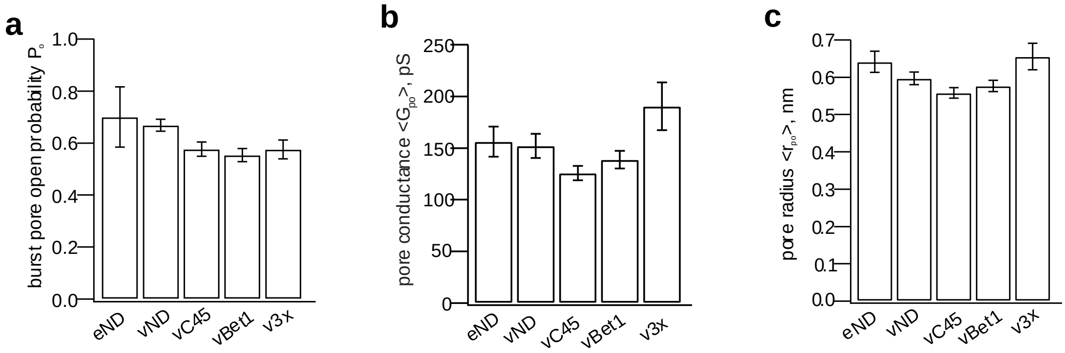

Supplementary Figure 12. a. Average pore open probability during a burst. **b**. Average open-pore conductances, $\langle G_{po} \rangle$. **c**. Average open-pore radii, $\langle r_{po} \rangle$. Number of patches tested for each condition is the same as in Fig. 4.

a

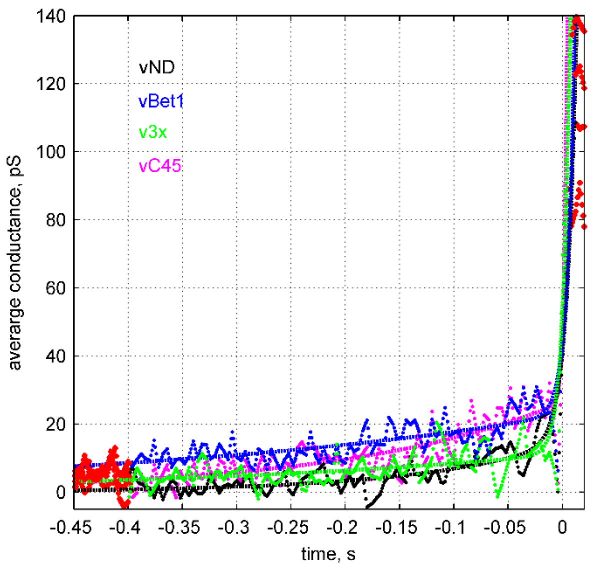

b

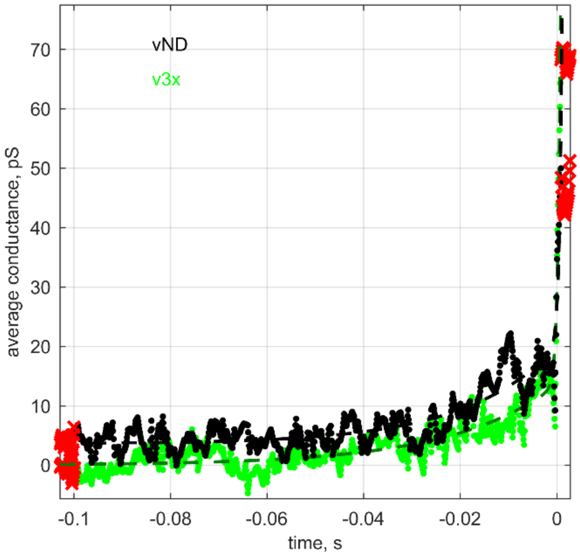

Supplementary Figure 13. Pore opening kinetics. **a**. Conductance traces were averaged over individual pores that were aligned to the first detected open sub-state as in Supplementary Figure 4 inset. The dashed black curve is a double exponential fit to the pore kinetics for vND-tCell data, $g(t) = a\exp(t/\tau_1) + b\exp(t/\tau_2)$, with best fit parameters (with 95% confidence bounds) $a = 7.65\ (6.60, 8.70)$, $b = 14.03\ (12.37, 15.69)$, $\tau_1 = 136\ (113, 160)$ ms, and $\tau_2 = 8.5\ (7.8, 9.3)$ ms ($R^2 = 0.920$). The other conditions yielded similar fits (dashed lines), with $\tau_1 = 140 - 400$ ms, and $\tau_2 = 3 - 9$ ms. The number of traces averaged were 122, 33, 64, and 68 for vND, v3xND, vC45ND, and vBet1ND, respectively. The red crosses indicate portions of the traces that were excluded from the fits. **b**. As in a, but for data with 10-fold improved time resolution (1.25 kHz bandwidth). Fast rise times estimated as in a were 0.5 ms for both vND and v3x samples, too fast to be quantified reliably even with improved time resolution.
